# Infection mechanisms and putative effector repertoire of the mosquito pathogenic oomycete *Pythium guiyangense* uncovered by genomic analysis

**DOI:** 10.1371/journal.pgen.1008116

**Published:** 2019-04-24

**Authors:** Danyu Shen, Zhaoyang Tang, Cong Wang, Jing Wang, Yumei Dong, Yang Chen, Yun Wei, Biao Cheng, Meiqian Zhang, Laura J. Grenville-Briggs, Brett M. Tyler, Daolong Dou, Ai Xia

**Affiliations:** 1 College of Plant Protection, Nanjing Agricultural University, Nanjing, China; 2 Department of Plant Protection Biology, Swedish University of Agricultural Sciences, Alnarp, Sweden; 3 Center for Genome Research and Biocomputing, Oregon State University, Corvallis, Oregon, United States of America; University College Dublin, IRELAND

## Abstract

*Pythium guiyangense*, an oomycete from a genus of mostly plant pathogens, is an effective biological control agent that has wide potential to manage diverse mosquitoes. However, its mosquito-killing mechanisms are almost unknown. In this study, we observed that *P*. *guiyangense* could utilize cuticle penetration and ingestion of mycelia into the digestive system to infect mosquito larvae. To explore pathogenic mechanisms, a high-quality genome sequence with 239 contigs and an N50 contig length of 1,009 kb was generated. The genome assembly is approximately 110 Mb, which is almost twice the size of other sequenced *Pythium* genomes. Further genome analysis suggests that *P*. *guiyangense* may arise from a hybridization of two related but distinct parental species. Phylogenetic analysis demonstrated that *P*. *guiyangense* likely evolved from common ancestors shared with plant pathogens. Comparative genome analysis coupled with transcriptome sequencing data suggested that *P*. *guiyangense* may employ multiple virulence mechanisms to infect mosquitoes, including secreted proteases and kazal-type protease inhibitors. It also shares intracellular Crinkler (CRN) effectors used by plant pathogenic oomycetes to facilitate the colonization of plant hosts. Our experimental evidence demonstrates that CRN effectors of *P*. *guiyangense* can be toxic to insect cells. The infection mechanisms and putative virulence effectors of *P*. *guiyangense* uncovered by this study provide the basis to develop improved mosquito control strategies. These data also provide useful knowledge on host adaptation and evolution of the entomopathogenic lifestyle within the oomycete lineage. A deeper understanding of the biology of *P*. *guiyangense* effectors might also be useful for management of other important agricultural pests.

## Author summary

Utilization of biocontrol agents has emerged as a promising mosquito control strategy, and *Pythium guiyangense* has wide potential to manage diverse mosquitoes with high efficiency. However, the molecular mechanisms underlying pathological processes remain almost unknown. We observed that *P*. *guiyangense* invades mosquito larvae through cuticle penetration and through ingestion of mycelia via the digestive system, jointly accelerating mosquito larvae mortality. We also present a high-quality genome assembly of *P*. *guiyangense* that contains two distinct genome complements, which likely resulted from a hybridization of two parental species. Our analyses revealed expansions of kinases, proteases, kazal-type protease inhibitors, and elicitins that may be important for adaptation of *P*. *guiyangense* to a mosquito-pathogenic lifestyle. Moreover, our experimental evidence demonstrated that some Crinkler effectors of *P*. *guiyangense* can be toxic to insect cells. Our findings suggest new insights into oomycete evolution and host adaptation by animal pathogenic oomycetes. Our new genome resource will enable better understanding of infection mechanisms, with the potential to improve the biological control of mosquitoes and other agriculturally important pests.

## Introduction

Mosquitoes are a major threat to global health since they are vectors of numerous devastating diseases, including malaria, dengue fever, Zika virus and other arboviruses, which together result in hundreds of millions of cases and several million deaths annually [1]. Existing commonly used control methods for reducing disease rely on the application of residual synthetic pesticides. However, intensive and repeated use of pesticides leads to ongoing development of resistance, environmental pollution and toxicity to human and non-target organisms [2]. Control strategies utilizing naturally occurring microbial pathogens have therefore emerged as a promising alternative. A particular focus has been on biological control agents [2]. Among them, the oomycete *Lagenidium giganteum* and the fungal pathogens, *Beauveria bassiana* and *Metarhizum anisopliae*, are well characterized, promising agents for mosquito larvae control, and have been produced commercially for field tests [2–4]. However, so far, available agents for mosquito control are rather limited.

Recently, a new mosquito-pathogenic oomycete, *Pythium guiyangense* X.Q. Su was isolated from infected larvae of *Aedes albopictus* from Guizhou, China [5]. It is a virulent pathogen of a wide range of mosquito larvae and is safe to non-target organisms [6, 7]. The pathogen also shows robust adaptability to a variety of natural environments and can be easily mass-produced [7]. All these properties of *P*. *guiyangense* make it of interest for practical applications as a potential mosquito control agent. However, little is known about the molecular mechanisms underlying the pathological processes on its mosquito hosts. It belongs to the genus *Pythium* (kingdom *Stramenopila*; phylum *Oomycota*, class *Peronosporomycetes*) [8]. Within the oomycetes, the genus *Pythium* is a genetically diverse group with a broad host range. For example, many *Pythium* species are important plant pathogens causing a variety of diseases [9, 10]. On the other hand, *P*. *undulatum* can infect fish and *P*. *insidiosum* is a well-known pathogen that is capable of infecting human and other mammals [11]. To date, only *P*. *guiyangense* has been proposed for mosquito control [5].

The availability of genome sequences of a variety of pathogenic oomycetes, including *Pythium* species, provides a unique opportunity for comparative analysis between *P*. *guiyangense* and other oomycetes with respect to the evolution of pathogenicity. A number of genome sequences of plant pathogenic oomycetes are now available [12–14]. In addition, the genomes of mycoparasitic *Pythium* species that infect fungi, the human pathogen *P*. *insidiosum*, and the fish pathogens *S*. *parasitica* and *S*. *diclina* have also been sequenced [15, 16]. Oomycetes secrete an arsenal of effectors into the host to manipulate the host immune system and enable parasitic infection [17]. These effectors have been a central question in the study of plant-oomycete interactions, including extracellular proteins such as toxins and hydrolases, and cell-entering proteins such as the RxLR (Arg-X-Leu-Arg) and Crinkler (CRN) effectors [18]. *P*. *guiyangense* may share conserved virulence effectors with other pathogenic oomycetes because of their close evolutionary relationships.

Here, we determined the infection cycle of *P*. *guiyangense* and demonstrated an unusual process, in which mycelia were devoured by the mosquito larvae and the mycelia inside digestive system could effectively initialize infection. Then we produced a high-quality genome sequence assembly, which represents the first draft genome from an insect pathogenic oomycete. Based on the transcriptome, effector prediction, and comparative genome analyses with other *Pythium* species, we investigated its insect-killing mechanisms and finally identified several cytoplasmic effectors having virulence functions in insect cells, thus expanding the roles of oomycete effectors.

## Results

### Two invasion pathways accelerate mosquito larvae mortality

The *P*. *guiyangense* isolate was reported to be highly virulent on mosquito larvae, but infection was not quantitated [5, 19]. We created a quantitative virulence assay by inoculating early second-instar larvae of *Aedes albopictus* or *Culex pipiens pallens* with *P*. *guiyangense* mycelia. The cumulative survival curves revealed that daily survival of *A*. *albopictus* and *Cx*. *pipiens pallens* larvae quickly declined from 3~4 days post-inoculation (dpi) onwards, reaching 76% mortality for *A*. *albopictus* and 69% for *Cx*. *pipiens pallens* by 10 dpi (S1A Fig). *A*. *albopictus* larvae died faster than *Cx*. *pipiens pallens* larvae, reaching 50% survival by day 6 compared to day 8 (S1A Fig). Furthermore, *P*. *guiyangense* could infect all the tested stages of *Cx*. *pipiens pallens*, including eggs, larvae, pupae and adults, resulting a visible accumulation of mycelia by 3–4 dpi (S1 Video) and all tissues were fully covered by mycelia (S1B Fig). Together, the results confirmed that *P*. *guiyangense* is highly efficacious in killing all life stages of *Aedes albopictus* and *Cx*. *pipiens pallens*.

**Fig 1:**
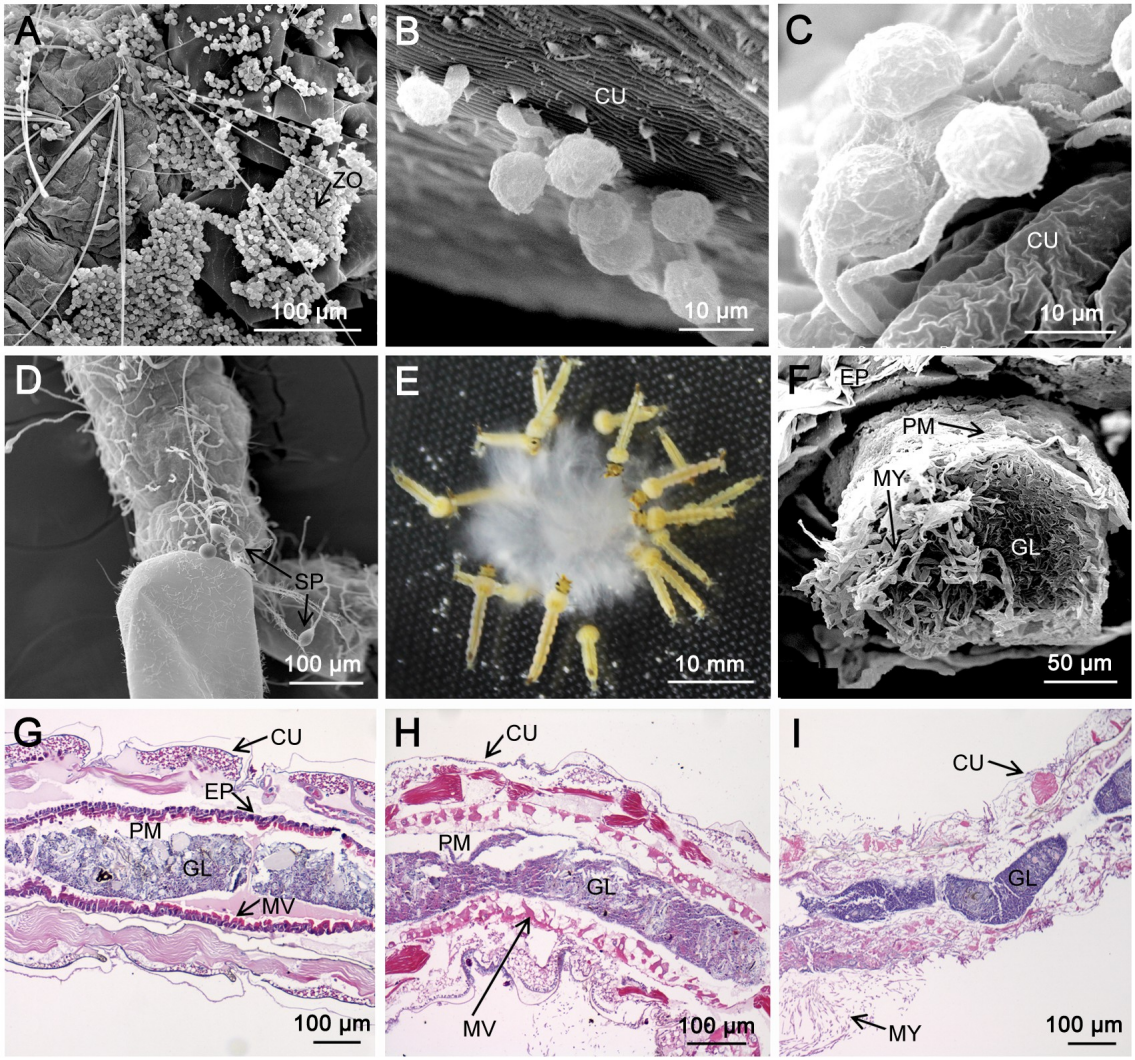
Two infection pathways by *P*. *guiyangense* into mosquito larvae. (A-D) Infection by direct penetration of the mosquito cuticle. (A) Zoospores gathered and adhered to the cuticle of a larva at 4 hpi. (B) Germinating cysts with germ tubes and appressoria at 8 hpi. (C) Germ tubes penetrating larvae at 12 hpi. (D) Sporangia produced at 24 hpi. (E-I) Infection by disruption of the mosquito digestive tract. (E) Mosquito larvae devouring *P*. *guiyangense* mycelia. (F) The mosquito larvae midgut packed with mycelia at 2 hpi. (G-H) The mosquito midgut epithelium, muscles, and connective tissues appeared disrupted at 12 hpi and 24 hpi. (I) Complete disruption of the mosquito epithelium and peritrophic membrane after 48 h. ZO: zoospore, MY: mycelia, SP: sporangia, CU: cuticle, EP: epithelium, PM: peritrophic membrane, GL: gut lumen, MV: microvilli.

To investigate the infection process of *P*. *guiyangense*, early second-instar larvae of *Cx*. *pipiens pallens* were incubated with mycelia or swimming zoospores. Our results showed that zoospores attached to almost any part of the mosquito larval cuticle (Fig 1A showing zoospore attachments on the thorax and abdomen**)**. Then, germination of the cysts occurred and appressorium-like swellings appeared at the tip of the germ tubes as visualized using scanning electron microscopy (SEM) (Fig 1B). Penetration hyphae emerging from the appressorium traversed the insect integument (Fig 1C) and invaded the hemocoel of larvae. Eventually the mycelia filled the whole body, then emerged through the inner cuticle and formed sporangia (Fig 1D). We also observed that *Cx*. *pipiens pallens* larvae readily ingested *P*. *guiyangense* mycelia even in an adequate food environment (Fig 1E, S1C Fig, S2 Video). A thick section of a moribund larva visualized with SEM showed that, following feeding, the midgut was completely packed with mycelia that could initialize infection (Fig 1F). After fixation, embedding in paraffin, and sectioning, microscopic observations showed that the midgut epithelium, muscles, and connective tissues appeared disrupted in the *P*. *guiyangense-*infected larvae (Fig 1G and 1H). After 48 hpi, infected larvae appeared almost devoid of internal organs or tissues and the whole body was permeated with mycelia (Fig 1I**)**. Thus invasion through the digestive tract is an effective route of infection by *P*. *guiyangense*. Taken together, these observations define two routes of invasion, namely infection through the exterior cuticle and through the digestive tract.

### General features of the genome and transcriptome sequences

A high quality genome sequence of *P*. *guiyangense* was generated using a hybrid strategy that combined sequences from Pacific Biosciences long reads and Illumina short reads. The genome assembly indicated in an estimated *P*. *guiyangense* genome size of 110 Mb and annotation predicted 30,943 protein-coding genes (Table 1). The assembled genome consisted of 239 contigs with an N50 contig length of 1,009 kb. To assess the completeness of the genome assembly, CEGMA analysis, which identifies orthologs of 248 ultra-conserved core eukaryotic genes (CEGs), was used to identify core genes in the *P*. *guiyangense* genome. The results revealed complete matches to 97.6% of CEGs and at least partial matches to 98.4% of CEGs within the *P*. *guiyangense* assembly; these results compared to only 78.2–94.4% of complete CEGs and 91.5–95.6% of partial CEGs in other *Pythium* genomes (S1 Table). Taken together, the comparison with other assembled *Pythium* genomes including *P*. *insidiosum*, *P*. *ultimum*, *P*. *aphanidermatum*, *P*. *arrhenomanes*, *P*. *irregulare* and *P*. *iwayamai*, revealed that the *P*. *guiyangense* assembly represented the best quality among the sequenced *Pythium* genomes so far.

**Table 1:** Genome statistics of *Pythium* genomes.

	*P*. *guiyangense*	*P*. *insidiosum*	*P*. *ultimum*	*P*. *aphanidermatum*	*P*. *arrhenomanes*	*P*. *irregulare*	*P*. *iwayamai*
Assembly size (Mb)	110.1	53.2	42.8	35.9	44.7	42.9	43.3
Contig/Scaffold Number	239	1192	975	5,667	10,978	5,887	11,542
N50 scaffold Length (Kb)	1009.1	146.3	773.5	37.4	9.8	23.2	11
G+C content (%)	58.3	57.9	49.8	51.4	54.6	53.4	53.1
Number of protein-coding genes	30,943	14,962	15,297	12,305	13,805	13,804	14,875
Average gene length (bp)	1658	2144	1299	1470	1339	1495	1325
Gene density (genes per Mb)	281	281	357	343	309	322	344
Introns per gene	2.0	2.4	1.6	1.3	1.6	1.7	1.8

Whole transcriptome sequencing (RNA-seq) was performed using Illumina sequencing of RNAs from *P*. *guiyangense* mycelia and from early second-instar larvae 24 hr after inoculation with *P*. *guiyangense* mycelia. Transcripts from each individual library matched approximately 69% of the genes, and together matched approximately 74% of the genes (S2 Table). A total of 3,354 genes (10.8%) were differentially expressed (>4-fold expression difference and statistical GFOLD value > 1 or < -1) between the two samples; 1,654 genes were up-regulated in the infection stage while 1,700 genes were down-regulated. Functional enrichment analysis revealed that genes encoding tyrosine kinase-like (TKL) kinases, subtilisin proteases, kazal-type protease inhibitors, and elicitin proteins, were over-represented among the differentially expressed genes (S3 Table). To validate the differentially expressed genes, we selected 18 genes that belonged to the above mentioned over-represented gene families and that were up- or down-regulated based on RNA-Seq data, and then measured their transcript levels by the qRT-PCR assay. The qRT-PCR results showed that the transcriptional patterns of 17 among the 18 genes were consistent with RNA-Seq results (S2 Fig), which further supported the general reliability of the RNA-Seq data.

### Interspecies hybridization may contribute to the large size of the *P*. *guiyangense* genome

**Fig 2:**
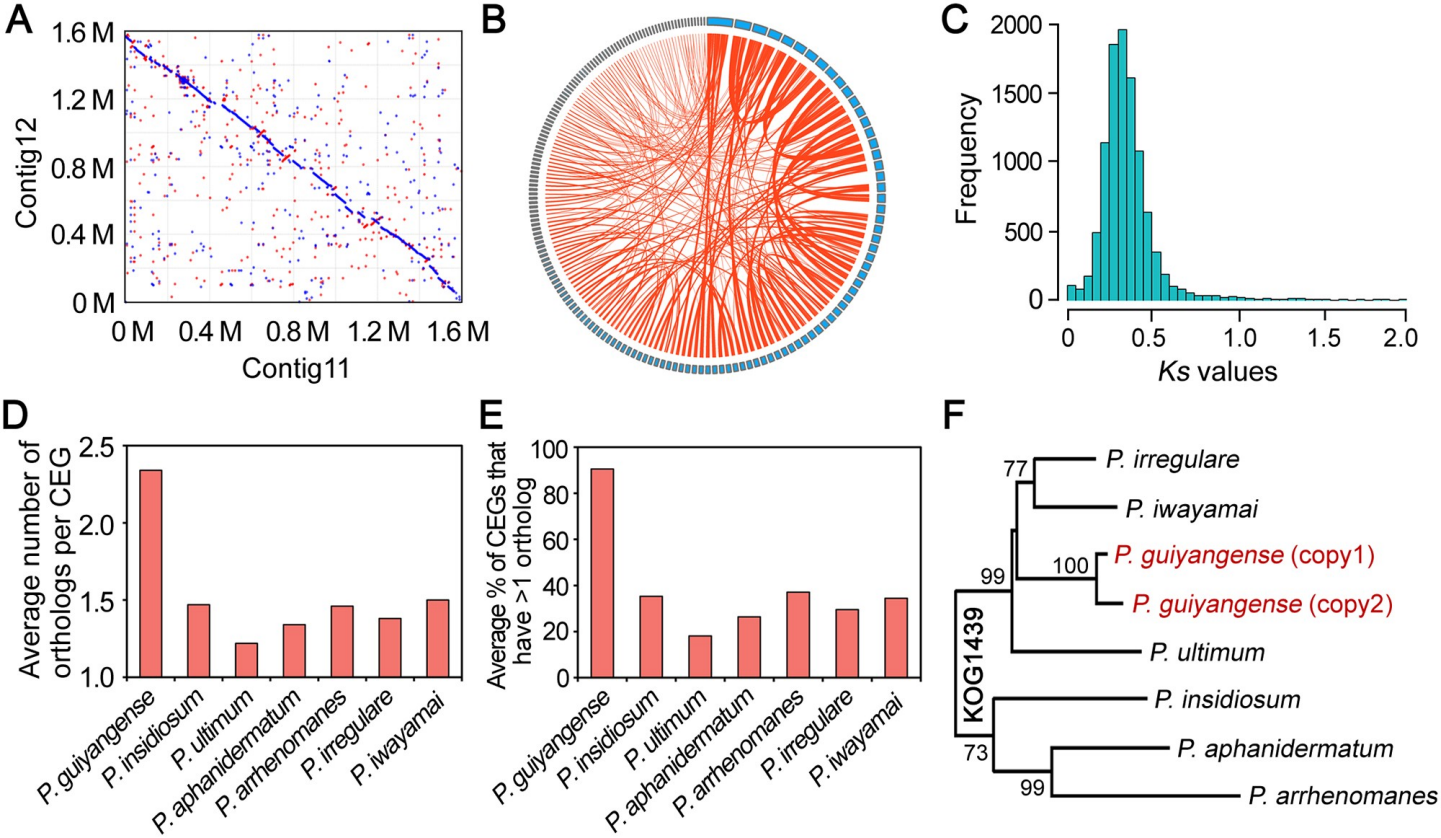
Interspecies hybridization may contribute to the large genome size. (A) Frequent synteny blocks identified between pairs of contigs in the *P*. *guiyangense* genome assembly. Shown is an example of nucleotide alignments of contig 11 (*x*-axis) versus contig 12 (*y*-axis). Each point in the plot corresponds to an aligned sequence of >500 bp. The red dots represent the same orientation and the blue dots represent reversed orientations. (B) Representation of homologous genes with conserved synteny in the genome. Each blue rectangle represents one contig. The circle represents all the contigs with synteny, and each line connects two homologous genes in the synteny block. Homologous genes which are found in the same contig were not drawn. The detailed information was listed in S4 Table. (C) The *Ks* distribution of paralogous gene pairs. (D) Comparison of the average number of orthologs for each CEG among *Pythium* species. (E) Comparison of the average percentage of detected CEGs that have more than one ortholog among *Pythium* species. (F) Phylogenetic analysis of one example of CEG named KOG1439 among *Pythium* species. This CEG had two copies in *P*. *guiyangense* while only one copy in each of other *Pythium* species.

Our comparative genome analysis revealed that the genome size (110 Mb) and predicted gene number of *P*. *guiyangense* (30,943 genes) were approximately twice those of the other sequenced *Pythium* species (Table 1). To explore the potential mechanisms underlying such a large genome size, we initially analyzed the repetitive DNA content within the *P*. *guiyangense* genome and found that the repeat sequence content was 6%, similar to that of *P*. *ultimum* (7%) which has a genome size of only 43 Mb, thus excluding the possibility that high repeat content was responsible for the large genome size, which was observed in *Ph*. *infestans* [14]. Previously, hybridization between two parental species has been reported in yeast and *Phytophthora* evolution [20, 21]. Most (84%) of the *Pythium* core genes (present in all the 7 *Pythium* genomes) were present in two copies in the *P*. *guiyangense* genome, consistent with a hybrid origin, which is similar to the yeast [20]. Typically, the copied core genes shared the highest sequence similarity with genes derived from the *P*. *irregulare* genome, however, the two copied genes were about 8% different in nucleotide sequence. To further confirm the hypothesis of hybridization, internal synteny is selected as a useful indicator, which could be used to evaluate hybridization between two parental genomes [22, 23]. Therefore, we characterized internal synteny across the *P*. *guiyangense* genome using the MCScanX program [24]. In the whole genome, 468 conserved synteny blocks with an average size of 192 kb were identified (Fig 2A, S4 Table). These synteny blocks covered 74% of all the contigs, and together spanned 84% of the genome, suggesting that the *P*. *guiyangense* genome could be classified into two subgenomes.

To further compare the genetic relatedness of the two parental subgenomes, the average nucleotide identity (ANI) was calculated, and the ANI between the two subgenomes revealed approximately 91% identity. Notably, a total of 11,068 pairs of homologous genes were identified in these synteny blocks (Fig 2B, S4 Table), consistent with a hybrid origin. We estimated the rates of synonymous substitutions per synonymous site (*Ks*) of 11,068 pairs of homologous genes. This analysis showed a synonymous site divergence peak of *Ks* = 0.35 (Fig 2C), indicating that the two subgenomes were relatively diverse. Taken together, we inferred that *P*. *guiyangense* was a hybrid genome derived from two distinct parental species.

To further investigate the parental species of *P*. *guiyangense*, we systematically analyzed CEGs in the 7 sequenced *Pythium* genomes. The majority of CEGs were present as two copies in the *P*. *guiyangense* genome but only one copy in each of the other *Pythium* genomes (Fig 2D and 2E). A total of 167 CEGs that contained 2 copies in *P*. *guiyangense* and also had orthologs in other *Pythium* species were utilized for phylogenetic analysis. For each phylogenetic tree, the two copies of the *P*. *guiyangense* CEG always clustered together most closely, and then clustered with the orthologs from the other *Pythium* species (one tree based on the KOG1439 protein is shown in Fig 2F as an example), indicating that the parental species of *P*. *guiyangense* were not represented in the data set. In addition, cytochrome oxidase II (*cox* II) and β-tubulin genes, which have been widely used as phylogenetic maker genes, were available in 35 *Pythium* species and contained 2 copies in *P*. *guiyangense*. Phylogenetic analyses of the two genes showed that the two *P*. *guiyangense* orthologs were more similar to one another than the nearest known species (*P*. *orthopogon*) (S3A and S3B Fig), indicating that the parental species of *P*. *guiyangense* were not represented based on current information. We speculate that the parental species of *P*. *guiyangense* are more closely related to each other than to the known *Pythium* species.

In parallel with the genome analysis, we noticed that an unusual high percentage of *P*. *guiyangense* zoospores contained two nuclei rather than one (S3C Fig). Among 500 observed zoospores, nearly 22% of them contained two nuclei in *P*. *guiyangense* while in *P*. *aphanidermatum* and *Ph*. *capsici*, all the spores had only one nucleus (S3C Fig). We then found that the *P*. *guiyangense* zoospores containing only one nucleus could also breed similar percent of zoospores containing two nuclei, and PCR amplifications resulted in presence of both of the two copied genes. This observation suggested that *P*. *guiyangense* might be a dikaryon, and its relationship with the complex genome is still under investigation.

### Evolutionary relationships and species-specific gene families

**Fig 3:**
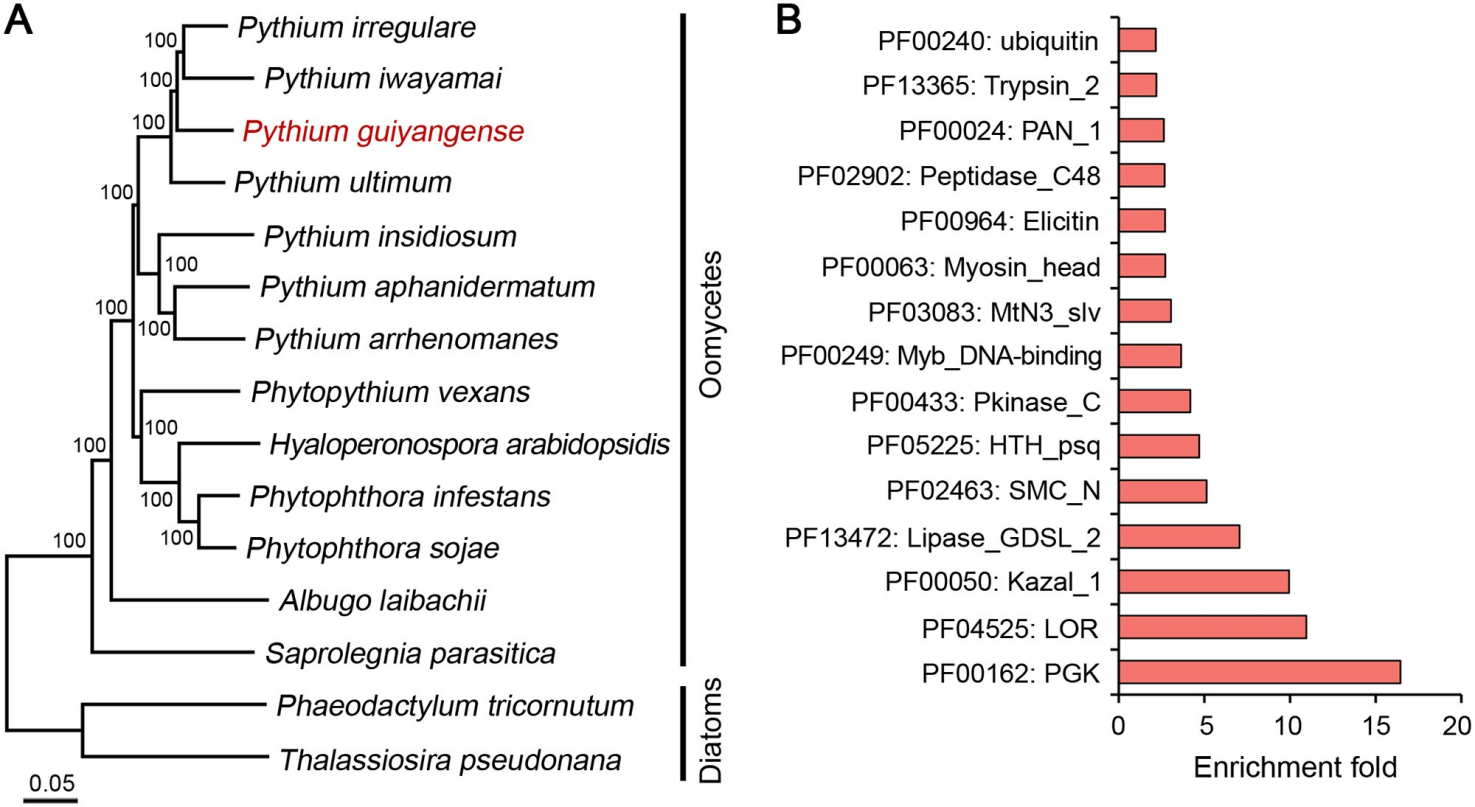
Phylogenetic relationships and species-specific genes. (A) Genome-based phylogenetic relationships of *P*. *guiyangense* and other oomycetes. The neighbor-joining tree was constructed from concatenated alignment of 248 core eukaryotic genes (CEGs) identified by CEGMA analysis. (B) Enrichment of PFAM domains in *P*. *guiyangense*-specific genes. The fold-enrichment (*y*-axis) corresponds to the frequency of a given PFAM domain in the species-specific genes divided by the frequency in the rest of the proteome.

To establish the phylogenetic relationship of *P*. *guiyangense* among oomycetes, a phylogenetic tree was constructed based on 248 CEGs from *P*. *guiyangense* and other 12 oomycetes, with the diatoms as outgroups (Fig 3A). The tree clearly showed that *P*. *guiyangense* was clustered within the clade formed by the plant pathogenic *Pythium* species, and was distantly related to other genera, including *Hyaloperonospora* and *Phytophthora*. This phylogeny was consistent with that in previous publications [15, 25]. These results imply that *P*. *guiyangense*, along with the mammalian pathogen *P*. *insidiosum* share common ancestors with the plant pathogenic *Pythium* species (Fig 3A).

To identify the genes responsible for host adaptation in *P*. *guiyangense*, the OrthoMCL tool was used to cluster the seven *Pythium* proteomes on the basis of protein sequence similarity. A total of 25,602 (83%) *P*. *guiyangense* genes had orthologs in other *Pythium* species. Among these, *P*. *guiyangense* shared 13,000 core genes with the other *Pythium* species. In addition, 5,341 genes were identified to be specific to *P*. *guiyangense* (S4 Fig). To gain insights into the features of species-specific genes in *P*. *guiyangense*, we compared the frequency of occurrence of protein family domains and identified highly over-represented domains included kinase (PF00433), kazal inhibitor (PF00050), elicitin (PF00964), and protease (PF02902) (Fig 3B, S5 Table). These gene families were also enriched among genes differentially expressed during infection.

### Expanded protein kinases involved in the infection process

**Fig 4:**
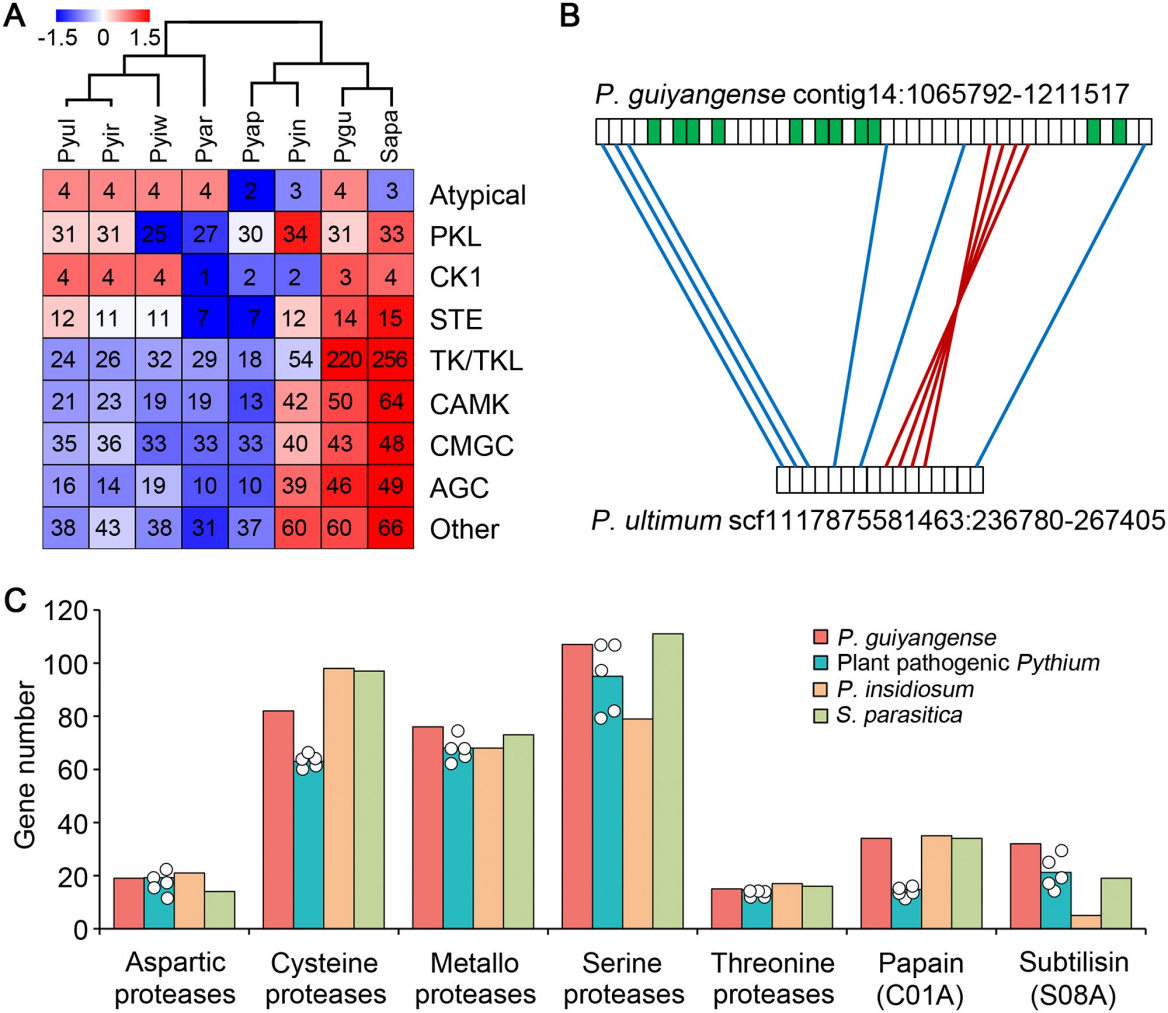
Expansion of kinases and proteases in *P*. *guiyangense*. (A) Comparison of kinase gene numbers between *P*. *guiyangense* and other oomycetes. The numbers of family members per haploid genome are shown in each case, including *P*. *guiyangense*. Overrepresented (white to red) and underrepresented (white to blue) numbers are depicted as Z-scores for each family. Pygu, *P*. *guiyangense*; Pyin, *P*. *insidiosum*; Pyap, *P*. *aphanidermatum*; Pyar, *P*. *arrhenomanes*; Pyir, *P*. *irregulare*; Pyiw, *P*. *iwayamai*; Pyul, *P*. *ultimum*; Sapa, *S*. *parasitica*. (B) Synteny of TKL kinase genes between *P*. *guiyangense* and *P*. *ultimum*. The region shown is an example of dense clustering of TKL kinase genes in the *P*. *guiyangense* genome assembly. The green boxes represent TKL kinase genes. Red lines join syntenic genes with the same orientation and the blue lines join genes with reversed orientations. (C) Comparison of protease gene numbers between *P*. *guiyangense* and other oomycetes. The teal columns represent the average number of genes per haploid genome of the five plant pathogenic *Pythium* species, while each dot represents the number in an individual plant pathogenic *Pythium* species. For *P*. *guiyangense*, the unique gene number in each haploid genome was used.

By searching with the HMM profiles of kinase domains derived from KinBase, 471 unique protein kinases (943 kinases in total) were identified in the *P*. *guiyangense* genome, greatly surpassing the numbers in plant pathogenic *Pythium* genomes, which range from 152 to 192 (Table 2). Intriguingly, other two animal pathogenic oomycetes, *P*. *insidiosum* and *S*. *parasitica*, also have expanded kinomes, coding for 286 and 538 kinases, respectively [15, 16]. We further classified the kinases into 9 families defined by Hanks and Hunter [26]. Five families, including TKL (tyrosine kinase-like), CAMK (calcium/calmodulin-dependent kinase), CMGC [including cyclin-dependent kinases (CDKs), mitogen-activated protein kinases (MAP kinases), glycogen synthase kinases (GSK) and CDK-like kinases], AGC (cAMP-dependent, cGMP-dependent and protein kinase C) and "other" were noticeably expanded in *P*. *guiyangense* (Fig 4A). Among the 5 expanded families, a total of 220 unique TKL genes were identified in *P*. *guiyangense* kinome. A comparison of the locations of TKL genes in the *P*. *guiyangense* and *P*. *ultimum* genomes revealed extensive rearrangements, which resulted from species-specific expansions at the locations of these genes (Fig 4B). Forty-six unique kinases belonging to AGC family and 50 unique members of the CAMK family were identified from *P*. *guiyangense*. Based on the RNA-Seq data, a total of 92 kinase genes were differentially expressed at the infection stage, including 52 TKL kinase genes (S6 Table). These results suggest that many of the protein kinases may be involved in regulation of infection processes and adaptation to the mosquito hosts.

**Table 2:** Protein families implicated in pathogen-host interactions.

	*P*. *guiyangense*^a^	*P*. *ultimum*	*P*. *irregulare*	*P*. *iwayamai*	*P*. *aphanidermatum*	*P*. *arrhenomanes*	*P*. *insidiosum*	*S*. *parasitica*
Kinases	471 (943)	185	192	185	152	161	286	538
Proteases	307 (615)	284	264	233	272	246	283	311
Serine proteases	109 (218)	108	97	79	108	83	79	111
Subtilisin proteases	32 (64)	30	19	18	25	14	5	19
Cysteine proteases	83 (167)	63	66	61	63	62	98	97
Papain proteases	34 (68)	17	16	14	14	13	35	34
Metallo proteases	79 (159)	75	66	63	68	68	68	73
Kazal protease inhibitors	19 (39)	11	10	8	12	9	27	5
Elicitins	10 (20)	0	2	0	0	0	0	0
Elicitin-like	45 (91)	40	25	23	23	29	50	29
NLP	1 (2)	7	4	4	4	5	0	0
CRN	19 (38)	46	10	33	45	46	44	0

^a^The numbers without brackets are numbers of unique genes per haploid genome, while numbers in the brackets are total gene numbers.

### A large number of proteases may facilitate cuticle penetration

Since major structural and physiological differences were observed between plant cell walls and insect cuticles, we compared the repertoire of plant cell wall and cuticle degrading enzymes encoded in the *P*. *guiyangense* genome to other oomycete genomes. Several groups of plant cell wall degrading enzymes, such as GH53, GH78, CE5, GH10 and GH11, and GH12 were completely absent in *P*. *guiyangense* (S7 Table). Genes encoding 12 unique pectin/pectate lyases (PL1, PL3 and PL4), two unique GH28 and 1 unique GH43 involved in pectin backbone degradation were identified in the *P*. *guiyangense* genome; however, RNA-Seq data showed that none of these genes exhibited up-regulation during mosquito infection processes.

*P*. *guiyangense* had more genes encoding proteases potentially involved in insect cuticle degradation than plant pathogenic *Pythium* species (Fig 4C). A total of 307 unique proteases (615 genes in total) were encoded in the *P*. *guiyangense* genome, compared to an average of 260 proteases in the plant pathogenic *Pythium* species (Table 2). The two animal pathogen genomes also had large numbers of proteases (Table 2, Fig 4C). Among them, genes encoding cysteine-, metallo- and serine-proteases were particularly highly expanded in *P*. *guiyangense* (Fig 4C). The subtilisin serine-protease family had the highest relative expansion with 32 unique genes in *P*. *guiyangense* (Table 2, Fig 4C). Phylogenetic analysis revealed that over half of the subtilisin proteases were recently expanded in *P*. *guiyangense* due to lineage-specific gene duplications (S5 Fig). Based on the RNA-Seq data, 31% of the total subtilisins were significantly up-regulated during mosquito infection. The peptidase_C1 and carboxypeptidases also exhibited significant expansion in *P*. *guiyangense* (Table 2, Fig 4C).

### Large family of kazal-type serine protease inhibitors (KPIs) potentially associated with infection

Protease inhibitors regulate various biological and physiological processes in all living systems as modulators of protease activity [27]. Among them, the kazal-type protease inhibitor (KPI) family is one of the best characterized [27]. A total of 19 unique kazal inhibitors were identified in the *P*. *guiyangense* genome, which exceeded those in plant pathogenic *Pythium* species (Table 2). The animal pathogen, *P*. *insidiosum* also encoded larger numbers of kazal inhibitors. A phylogenetic tree was constructed using the *Pythium* kazal inhibitors, and the majority of genes derived from *P*. *guiyangense* and *P*. *insidiosum* formed clusters that were species-specific (S6A Fig), implying that these genes were retained and diversified independently in these two animal pathogens. During mosquito infection, 25% of the *P*. *guiyangense* kazal inhibitors were up-regulated, and four of these exhibited transcript levels over 40 times those in the mycelia sample (S6B Fig). The transcriptional patterns of the 4 kazal inhibitors at three infection time points were analyzed by qRT-PCR, and results revealed that all the 4 genes were up-regulated during the infection process (S6C Fig). Further analysis demonstrated that all of the up-regulated kazal inhibitors contained signal peptides, indicating that they could play important roles in the pathogenesis.

### Elicitor genes and host detection

**Fig 5:**
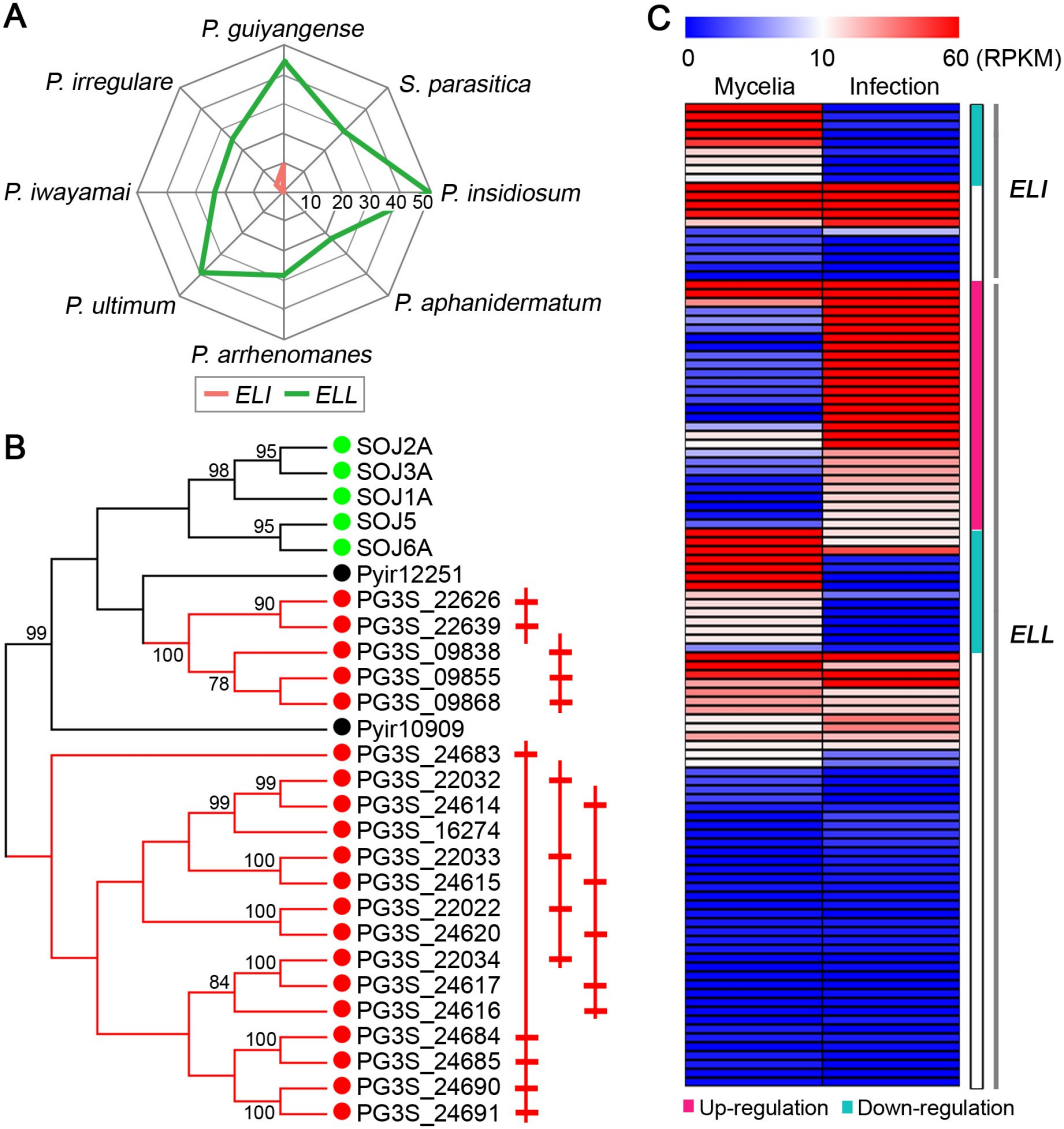
Expansion of *ELI* and *ELL* genes. (A) Radar charts showing the numbers of elicitin (*ELI*) and elicitin-like (*ELL*) genes found in each *Pythium* or *S*. *parasitica* genome. For *P*. *guiyangense*, the unique gene number in each haploid genome was used. Each spoke in the chart represents one species. The concentric circles form a ruler with a primary unit of 10 genes. (B) Phylogenetic tree of the *ELI* genes, showing species-specific expansion of *P*. *guiyangense ELI* genes. Members from *P*. *guiyangense* (PG3S, red), *P*. *irregulare* (Pyir, black), and *Ph*. *sojae* (SOJ, green) are included. On the right the physically linked ELI genes are schematically drawn. (C) Relative transcript abundance of *ELI* and *ELL* genes at 24 hpi infection versus mycelia tissue. The teal bar highlights the significantly down-regulated genes, and the pink bar highlights the significantly up-regulated genes.

A common feature of many plant pathogenic oomycetes is the secretion of a variety of apoplastic (extracellular) proteins to promote infection, some of which can be detected by the host immune system. These include elicitins (ELIs; lipid-binding proteins), elicitin-like (ELL) proteins and Nep1-like proteins (NLP) [28]. Ten unique *ELI* genes were identified in the *P*. *guiyangense* genome. In contrast, only 2 *ELI* genes were found in the *P*. *irregulare* genome, and none were identified in the other *Pythium* and *S*. *parasitica* genomes (Fig 5A, Table 2). Based on phylogenetic analysis of the elicitin domains, *P*. *guiyangense ELIs* were distributed into two clades, and one clade included genes from diverse species while the second clade only contained *P*. *guiyangense ELIs* (Fig 5B). Moreover, 19 of the 20 *ELI* genes were physically clustered in the *P*. *guiyangense* genome, suggesting that *ELIs* were expanded in a species-specific manner. In contrast to the *ELI* genes, *ELL* genes were widely distributed in all the detected *Pythium* genomes. Both *P*. *guiyangense* and *P*. *insidiosum* had more *ELL* genes (45 and 50 unique genes) than the plant pathogenic *Pythium* species (23–40 genes) (Fig 5A, Table 2). Further phylogenetic analysis showed that over half of the *ELL* genes were distributed in nine clades which were specific to *P*. *guiyangense* and contained at least four members; thus many *ELL* genes were specifically expanded in *P*. *guiyangense*. Based on the *P*. *guiyangense* transcriptome analysis, 45% of the *ELI* genes were differentially expressed, and all were down-regulated during infection (Fig 5C). In contrast, 31% of the *ELL* genes were up-regulated while 15% were down-regulated during infection (Fig 5C). This observation suggested that the diverse *ELIs* and *ELLs* had a variety of different functions relative to growth and infection.

Another common apoplastic effector family is the necrosis and ethylene-inducing-like proteins (*NLP*) genes. Many NLPs, but not all, can trigger cell death and defense responses in plants [29]. Only 1 unique *NLP* gene was found in *P*. *guiyangense* and none were found in *P*. *insidiosum* (Table 2). This NLP protein belonged to type 1 NLP subfamily with two conserved cysteine residues. Transcriptional analysis revealed no significant change during infection. These observations suggest that NLP proteins may not participate in oomycete-animal interactions.

### Some CRN effectors can induce insect cell death

Crinkler (CRN), a large class of cytoplasmic effectors, was first identified in *Ph*. *infestans* as a family of proteins that could cause plant cell death and defense responses [30]. A total of 38 CRN candidates were predicted in *P*. *guiyangense* (S8 Table), compared to 10–46 predicted CRN proteins in the other *Pythium* species using the same method (Table 2). Examination of protein alignments of *P*. *guiyangense* CRN effectors revealed considerable conservation of the characteristic LxLFLAR/K and HVLVxxP motifs, which were similar to those observed in plant pathogenic *Pythium* species [13, 25]. Based on five secretion signal predictors, 74% of CRN candidates in *P*. *guiyangense* contained a potential signal peptide or non-classical secretion signal (S8 Table), suggesting that the majority of *P*. *guiyangense* CRN proteins might be secreted into mosquito hosts.

**Fig 6:**
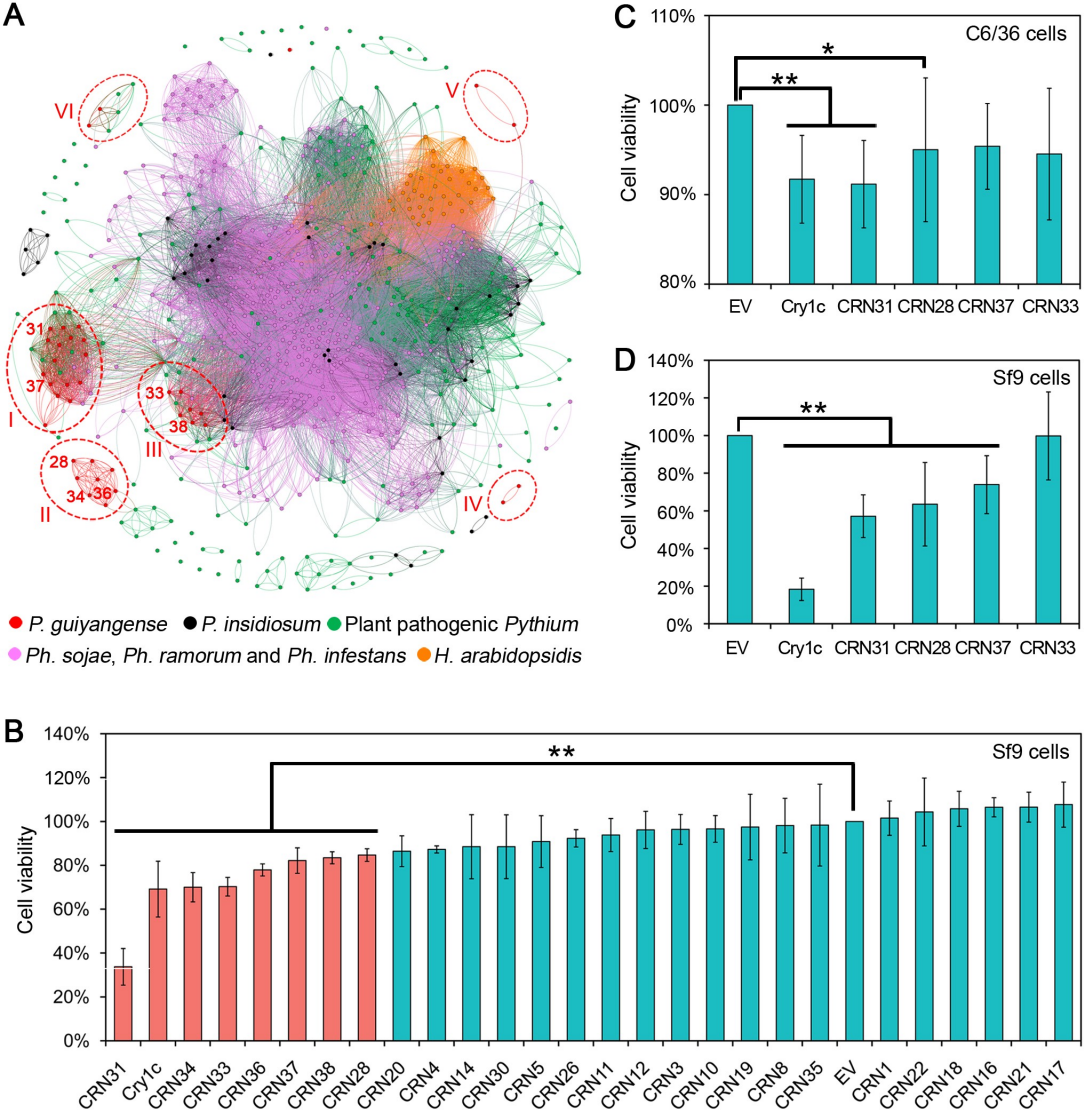
Diversification of *P*. *guiyangense* CRN proteins and their potential contributions to virulence. (A) Homology network of oomycete CRN proteins. Each protein is represented by a node. An edge joining two nodes represents sequence similarity shared by the two proteins. The red circled clusters (cluster I-VI) represent *P*. *guiyangense* specifically expanded CRN proteins. The number on the left of the red node represents *P*. *guiyangense* CRN protein ID. For example, 31 represents CRN31. (B) Evaluation of CRN protein toxicity to *Spodoptera frugiperda* cells (Sf9). The Sf9 cells were transfected with DNA encoding the CRN proteins. The protein toxicity was measured as the remaining number of viable cells compared to the empty vector (EV) negative control. Protein expression was assessed by western blots (S7A Fig). (C-D) Assay of CRN protein toxicity to insect cells (C, mosquito C6/36 cells; D, Sf9 cells) using CRN proteins produced in *E*. *coli* cell extracts. CRN protein production was assessed by western blots (S7C Fig). Differences were assessed for significance using Student’s t-test: *, P<0.05 and **, P<0.01.).

A homology network of the oomycete CRN proteins was generated to investigate the evolutionary relationships between *P*. *guiyangense* and other oomycetes. The network is composed of 633 nodes in which each node represents an individual CRN protein. The network contains 34,149 edges that link nodes if the node proteins are homologous based on an all-versus-all BlastP search with an E-value cutoff of 10^−10^. As shown in Fig 6A, the network was comprised of a crowd of disconnected clusters and a small number of singletons. *P*. *guiyangense* CRN proteins were mainly distributed in 3 large and 3 small clusters (cluster I-VI represented by red dotted circles). Cluster I and III were composed primarily of *P*. *guiyangense* CRN proteins, with some of these proteins having homology to *Pythium* proteins. Notably, cluster II, IV and V only contained CRN proteins derived from *P*. *guiyangense*, revealing that these CRN proteins did not share significant sequence similarity with other oomycete CRN proteins. Moreover, all of the *P*. *guiyangense* CRN proteins showed sequence divergence of at least 50% with the most similar CRN protein in any plant pathogenic *Pythium* species, indicating that the CRN proteins are highly divergent between insect pathogenic *Pythium* species and plant pathogenic *Pythium* species.

To explore the possible functions of *P*. *guiyangense* CRN proteins in insect cells, twenty-six *CRN* genes were expressed in *Spodoptera frugiperda* cell (Sf9) lines; successful expression of the proteins was confirmed with western blots or by detecting fluorescence signals under the fluorescence microscope (S7A and S7B Fig). The cell counting Kit-8 assay was used to determine protein toxicity to cells, with the *Bacillus thuringiensis* Delta-Endotoxin Cry1C as a positive control [31]. The results showed that 7 CRN proteins (CRN31, 33, 34, 36, 37, 38, and 28) significantly decreased the viability of Sf9 cells while the remaining CRN proteins produced responses similar to the negative control (Fig 6B). Notably, CRN31 appeared to be the most toxic to Sf9 cells. To further validate the toxicity of these 7 CRN proteins, a prokaryotic expression system was used to obtain recombinant CRN proteins (S7C Fig). *E*. *coli* crude extracts containing the expressed proteins were then incubated with Sf9 cells and with mosquito *Aedes albopictus* C6/36 cells, respectively, to determine the toxicity using the cell counting Kit-8 assay. The results showed that CRN31 and CRN28 significantly reduced the viability of Sf9 cells and C6/36 cells (Fig 6C and 6D). Since the transcript levels of the *CRN31* and *CRN28* genes were not elevated during infection at 24 hpi as measured by RNA-seq (S8 Table), we used qRT-PCR to test whether the two *CRN* genes were significantly up-regulated during earlier infection stages (1–4 hpi) (S7D and S7E Fig). *CRN31* exhibited the highest transcript level change with a 65 fold change at 2 hpi while CRN28 showed 4–5 fold changes at 1–3 hpi (S7D and S7E Fig). Together these results suggested that CRN31 and possibly CRN28 might act as cell-killing effectors during insect infection.

## Discussion

In this study, we have determined the mode of infection of *P*. *guiyangense* on mosquito larvae. In our experiments, *P*. *guiyangense* caused up to 76% mortality for *A*. *albopictus* and 69% for *Cx*. *pipiens pallens* larvae. Analogous to most of the entomopathogenic fungi, *P*. *guiyangense* hyphae emerging from germinating zoospore cysts entered their host directly through the exterior cuticle, propagated inside hosts, and produced sporangia to start a new cycle of infection. Another infection route of *P*. *guiyangense* was through the ingestion of mycelia by larvae. Mycelia in the digestive tract progressively destroyed internal tissues of the larval midgut, leading to host death. The most common invasion route for aquatic insect pathogens, including *Metarhizium anisopliae*, *Aspergillus clavatus* and *Beauveria Bassiana*, was through ingestion of spores to infect their host [4, 32, 33]. *P*. *guiyangense* has evolved a similar strategy to initiate infection in the digestive system. Overall, *P*. *guiyangense* utilizes cuticle penetration and ingestion of mycelia into the digestive system to infect mosquito larvae. We speculate that firm adhesion of zoospores to the mosquito larvae epicuticle is critical for the success of the *P*. *guiyangense* pathogen which involves a combination of passive hydrophobic and electrostatic forces as well as protein interaction. Hydrophobins found in the outer layer of the spore cell wall of *Beauveria Bassiana*, mediate adhesion to the arthropod cuticle [34, 35]. Hydrolytic enzymes, Mad1 and Mad2, also assisted in attachment of the fungi to insects [36]. To identify the factors that promote attachment and ingestion of *P*. *guiyangense* by the mosquitoes would be interesting to further explore in the future.

To probe the molecular basis underlying the interactions of *P*. *guiyangense* with insects, a high-quality genome assembly and transcriptome sequences were generated for *P*. *guiyangense*. Our results reveal that *P*. *guiyangense* is probably a hybrid genome derived from two parental species. Natural interspecies hybridization events have been described in the genus *Phytophthora* such as *Ph*. *andina*, *Ph*. *nicotianae* and *Ph*. *cactorum* [37, 38]. It is believed that interspecies hybridization has the potential to create new strains that have a new or expanded host range [21]. Considering the distinct hosts, we speculate that the hybrid feature of *P*. *guiyangense* contributes to its adaptation of the mosquito host. The two parental subgenomes of *P*. *guiyangense* are approximately 9% different in nucleotide sequence, suggesting that the two parents are relatively diverse, however, the potential parents are still mysterious based on limited *Pythium* data. We will pay close attention to the new information of *Pythium* and update the concerns in future study.

The phylogenetic analysis of the currently sequenced oomycete pathogens together with two diatoms demonstrated *P*. *guiyangense* is closely related to three plant pathogenic *Pythium* species (*P*. *irregulare*, *P*. *iwayamai* and *P*. *ultimum*) but has a slightly more distant relationship with the mammalian pathogen, *P*. *insidiosum*. This finding suggested that as a facultative mosquito pathogen, *P*. *guiyangense*, may have evolved from a common ancestor with the plant pathogens. This result is highly concordant with recent analysis indicating the mosquito oomycete pathogen, *L*. *giganteum* has also evolved from a plant pathogen [3].

In conjunction with the transcriptome analysis, oomycete genome comparisons identified several gene families that might contribute to *P*. *guiyangense* virulence. In this study, 471 putative unique kinases (943 kinases in total) were identified in the *P*. *guiyangense* genome. Comparison with other sequenced oomycete genomes revealed that the genomes of the animal pathogens, *P*. *guiyangense*, *P*. *insidiosum* and *S*. *parasitica*, also encoded significantly more kinases than the plant pathogenic *Pythium* genomes [15, 16]. Transcriptome analysis revealed that a total of 92 kinases were differentially expressed during infection of *P*. *guiyangense* against mosquito larvae, implying that protein kinases may be involved in regulating virulence. We also found that genes involved in insect cuticle degradation were expanded in *P*. *guiyangense* while proteins for plant cell wall penetration were absent or lost functions. The *P*. *guiyangense* genome encoded a significantly larger number of proteases than plant pathogenic *Pythium* species, including cysteine-, metallo-, and serine-proteases. Transcript levels of 31% of the total subtilisin-like serine proteases were significantly elevated when *P*. *guiyangense* invaded mosquito larvae. Some of these proteases were reported as key virulence determinants in entomopathogenic fungi [39], supporting a potential role of these proteases in *P*. *guiyangense* infection.

A large number of Kazal proteinase inhibitors (KPIs) were characterized from *P*. *guiyangense* and 25% of these genes were up-regulated during infection of mosquito larvae, suggesting KPIs may be involved in pathogenicity. Our study demonstrated that one invasion route of *P*. *guiyangense* was through ingestion of mycelia in the digestive system. The mosquito midgut contains an abundant array of secreted serine proteases for digestion, providing nutrition for development [40, 41]. To aid in colonization in its hosts, *P*. *guiyangense* may secrete protease inhibitors, such as KPIs for protection from these proteolytic enzymes. This is consistent with previous studies which show that the animal parasite, *Toxoplasma gondii* secretes TgPI-1 and TgPI-2, and Hookworm, *Ancylostoma ceylanicum*, secretes a 8-kDa broad spectrum serine protease inhibitor of the Kunitz family into the host digestive tract to aid in infection [42–44]. Serine proteases are also key components of immune responses and KPIs may manipulate host immune defenses for pathogenicity [45]. A kazal-like serine protease inhibitor was characterized from the plant pathogenic oomycete, *Ph*. *infestans*, and it targeted protease P69B to counteract tomato defense responses [46]. Another oomycete pathogen, *Ph*. *palmivora* also produced a KPI, PpEP to suppress plant defense [47]. Therefore, we speculate that the large number of KPIs secreted by *P*. *guiyangense* may suppress mosquito immune defenses by targeting serine proteases.

In addition to hydrolytic enzymes, plant pathogenic oomycetes deliver a diverse battery of other secreted proteins into host tissue to support infection and interfere with host immune responses, including lipid-binding proteins (elicitins), toxins (e.g. NLPs), and host-cell-entering RxLR and CRN effectors [48, 49]. We found distinct sequence and evolutionary features of these proteins in *P*. *guiyangense*. Firstly, no statistically significant evidence for RxLR effectors encoded in the *P*. *guiyangense* genome was found, in agreement with previous reports [13, 25]. Secondly, 10 unique *ELI* genes were present in the *P*. *guiyangense* genome whereas these genes were largely absent from the other *Pythium* species, including *P*. *insidiosum*. The *P*. *guiyangense ELI* genes appear to have expanded relatively recently to form two species-specific clades, and one clade appears to share a common origin with the *Ph*. *sojae* genes. In contrast to *ELIs*, *ELLs* have been widely found in all sequenced *Pythium* species. RNA-Seq data showed that *ELI* and *ELL* genes show differential expression patterns in *P*. *guiyangense*. *ELI* genes were typically highly expressed in the mycelia stage while a large number of *ELL* genes were up-regulated in the infection stage. These results suggested that the two subclasses of elicitins may be involved in different functions. Elicitins have also been reported in another mosquito pathogenic oomycete, *L*. *giganteum* [3], suggesting that elicitins in the two aquatic insect oomycetes may be linked to pathogenicity towards the insect hosts.

CRN effectors are considered more ancient cytoplasmic effectors than RxLRs, as they are distributed across a wide range of oomycetes [14, 25] and have also been reported in the fungal animal pathogen, *Batrachochytrium dendrobatidis* [50] and in arbuscular mycorrhizal fungi [51]. Interestingly, CRN proteins were also detected in the mosquito pathogenic oomycete *L*. *giganteum* [3, 52]. They are presumed to enter the host cytoplasm and manipulate cell death and defense responses [30, 53]. It has been widely reported that only a handful of oomycete CRN proteins were predicted to contain canonical signal peptides [13, 14]. In this study, four different signal peptide predictors and one non-classical secretion signal predictor were used, and the majority (74%) of *P*. *guiyangense* CRN proteins were predicted to contain potential secretion signals, suggesting that these CRN proteins very likely were secreted into mosquito hosts. Once inside host tissue, the roles of these putative effectors in animal pathogenic oomycetes remains unclear. One investigation detected CRN effectors in an entomopathogenic oomycete, *Lagenidium giganteum*, but their roles in the mosquito pathogenicity process remained unclear [52]. In this study, twenty-six CRN candidates were characterized in *P*. *guiyangense* and insect cell line transformation experiments revealed that CRN31 and CRN28 were toxic to *Spodoptera frugiperda* (Sf9) cells and to a lesser extent to *Aedes albopictus* (C6/36) cells. Therefore, we speculate that *P*. *guiyangense* has evolved distinct lineages of CRN effectors that are secreted into mosquito cells as virulence factors to induce host cell death.

Overall, we have demonstrated that two infection routes are available for infection of mosquitoes by *P*. *guiyangense*. The high-quality genome sequence of *P*. *guiyangense* provides new insights into study oomycete evolution and host adaptation because it is an oomycete pathogen that has adapted to mosquitoes. Genome comparisons suggest adaptations to a mosquito-pathogenic lifestyle include loss of plant cell wall degrading enzymes and NLP proteins, and expansions of kinases, proteases, and kazal-type protease inhibitors. Oomycete intracellular CRN effectors were identified and insect cell toxicity was identified in at least one of them, which could serve as a new resource to control agricultural important pests.

## Materials and methods

### Strain and mosquito source

The *P*. *guiyangense* strain Su was kindly provided by Dr. Xiaoqing Su from Guiyang Medical University, Guiyang, China and was maintained on 10% vegetable juice (V8) medium in the dark at 25 ± 1°C. The Nanjing laboratory strains of *Aedes albopictus* and *Cx*. *pipiens pallens* were obtained from Jiangsu Provincial Center for Disease Control and Prevention, Nanjing, China, and were kept at a temperature of 25 ± 1°C in a 16L: 8D photoperiod.

### Pathogenicity assays and infection processes of *P*. *guiyangense*

For mycelia infection assays, tests were carried out in plastic cups (capacity of 200 mL), each containing 25 early second-instar larvae and 4 agar plugs (10 mm × 10 mm in size) of *P*. *guiyangense* mycelia in 100 mL of deionized distilled water. The numbers of dead larvae were recorded every 24 hours for 10 days and each treatment was replicated at least three times. For zoospore infection assays, zoospores were prepared according to the method previously described [54], and then batches of 25 early second-instar larvae were exposed to a concentration of 10^7^ zoospores ml^-1^ in individual cups to examine the cuticle penetration process. The progress of infection in the larvae was initially documented using light microscope every 2 h for 48 h. For scanning electron microscopy (SEM), representative larvae were collected at 0.5, 2, 4, and 48 hpi and fixed in 2.5% glutaraldehyde solution. The fixed larvae were then rinsed three times in 0.1 M PBS, dehydrated sequentially in 30%, 50%, 70%, 80%, 95% and 100% ethanol, subjected to critical point drying, mounted, and finally gold coated for viewing. To investigate the digestive system infection, larvae were inoculated with 4 agar plugs (10 mm × 10 mm in size) of *P*. *guiyangense* mycelia. After different times post-inoculation, larvae were examined with SEM as described above or else embedded in paraffin, sectioned, and stained with haematoxylin and eosin for light microscope observation.

### Genome assembly

High-quality genomic DNA of *P*. *guiyangense* was prepared and submitted for genome sequencing using the PacBio and Illumina NGS platforms by Berry Genomics Corporation. The 450-bp paired-end libraries were constructed and sequenced on the Illumina HiSeq 2500 platform. The resultant short reads were processed to remove adapter sequences and low-quality sequences, resulting in 10.32 Gb of clean data (approximately 100-fold coverage). Two PacBio 20-kb SMRTcell libraries were constructed and sequenced on the Sequel platform. The generated raw reads were filtered by trimming the adapter sequences and low-quality sequences. This produced 7.55 Gb of cleaned sequences, with an average cleaned read length of 7.12 kb (approximately 70-fold coverage). Both the Illumina and PacBio SMRT sequencing data were used for the genome assembly. The *de novo* assembly was produced using the PacBio Hierarchical Genome Assembly Process HGAP 3.0 program [55]. First, the PacBio SMRT sequence data were error-corrected using the long filtered read and sub-read cyclic consensus sequences using HGAP error correction. The error-corrected long reads were then assembled using HGAP with default parameters. The Illumina paired-end reads were aligned to the PacBio assembly with BWA [56], and paired-end reads with concordant alignments were selected with SAMtools view for error correction. A final genome assembly error correction was conducted using the Pilon tool [57]. This Whole Genome Shotgun project has been deposited at DDBJ/ENA/GenBank under the accession QXDM00000000.1. The CEMGA pipeline was used to evaluate the completeness and continuity of the genome on the basis of 248 core eukaryotic genes [58].

### Gene prediction and functional annotation

The *P*. *guiyangense* assembly was masked for low complexity, as well as known transposable elements using RepeatMasker (www.repeatmasker.org). Genes in the repeat-masked genome were predicted using two predictors, AUGUSTUS [59] and SNAP [60]. The *P*. *guiyangense* core eukaryotic genes identified by CEGMA analysis were used to train the gene predictor SNAP. The AUGUSTUS predictor was trained using *P*. *ultimum* proteins. SNAP and AUGUSTUS were then used as a part of the MAKER software to conduct the gene prediction. Protein sequences from six sequenced *Pythium* species, plus *Ph*. *sojae*, *Ph*. *infestans*, *H*. *arabidopsidis* and *S*. *parasitica* were submitted to MAKER as extrinsic sources of gene structure evidence to improve sensitivity of gene predictions. The transcripts discovered based on the RNA-Seq data were also submitted to MAKER as EST evidence. MAKER was set to filter out short gene models that produce proteins with fewer than 30 amino acids.

All the protein sequences from *P*. *guiyangense* were searched against themselves using the BlastP program with the E-value setting to 10−^10^. Then, the BlastP result file and the GFF file of the *P*. *guiyangense* genome were inputted into the MCScanX program to analyze the synteny blocks and homologous genes located in synteny blocks [24]. Circular representations of these homologous genes were produced using Circos program [61]. The *Ks* values of each pair of homologous genes were calculated using KaKs_Calculator 2.0 [62].

Whole genome protein families were classified by Pfam analysis [63]. The proteomes were screened for CAZymes (carbohydrate active enzymes) using Hmmscan from the HMMER package and the dbCAN HMM profile database [64]. Putative proteases and protease inhibitors were identified by using batch BLAST at the MEROPS server [65]. Protein kinases were classified by Hmmsearch against KinBase (www.kinase.com).

### Transcriptome analysis

A sample of 30 early second-instar larvae inoculated with *P*. *guiyangense* mycelia was collected at 24 hpi as the infection stage, and another sample of *P*. *guiyangense* mycelia was harvested as the control. There were no biological replicates. The total RNAs of the two samples were extracted according to the method previously described [54], and then sequenced by Berry Genomics Corporation using the Illumina 2500 platform. The 150 bp paired-end reads were filtered for quality as described above and aligned to the *P*. *guiyangense* genome assembly using Tophat with a maximum of two mismatches [66]. The mapped reads were quantified using the Cufflinks program [67], and the transcript level of each gene was quantified as RPKM (reads per kilobase transcript length per million reads mapped). Differentially expressed genes were identified using the GFOLD algorithm [68]. GFOLD was developed for unreplicated RNA-Seq data and assigns statistics for expression changes based on the posterior distribution of log fold change. Genes with four-fold change and GFOLD > 1 or < -1 were considered differentially expressed between two samples. The reads were also assembled *de novo* using the Trinity package [69] with default settings to serve as additional evidence for gene prediction.

To detect the transcript levels of particular *P*. *guiyangense* genes during infection stages, qRT-PCR assays were performed. The samples of early second-instar larvae inoculated with *P*. *guiyangense* mycelia at different infection time points were collected, and another sample of *P*. *guiyangense* mycelia was harvested as the control. Then the total RNAs of the above samples were extracted for qRT-PCR assay. qRT-PCR was performed using an ABI Prism 7500 Real-Time PCR system (Applied Biosystems) with SYBR Premix Ex Tag according to the manufacturer’s instructions. The comparative threshold cycle (Ct) method was used to determine relative transcript levels through ABI 7500 System Sequence Detection Software. The relative transcript levels of particular *P*. *guiyangense* genes were normalized to the mycelia data using the *actin* gene as internal standard. At least three biologically independent replicates of the qRT-PCR experiments were carried out.

### Orthology and phylogenetic analysis

A phylogenetic analysis was conducted on the core eukaryotic genes identified using the CEGMA pipeline. Multiplex sequence alignments of proteins were made with ClustalW [70] and subsequently concatenated. A neighbor-joining tree was built using MEGA5 with 1000 fold bootstrapping for distance estimation [71]. Orthologous and paralogous groups among the seven *Pythium* genomes were determined using OrthoMCL with default parameters: BLASTp E-value cutoff of 10^−5^ and inflation index of 1.5 [72]. The output of OrthoMCL was parsed to separate core, conserved and specific clusters. Pfam domain enrichment analysis was undertaken on genes that were specific to *P*. *guiyangense*. The fold-enrichment corresponds to the frequency of a given PFAM domain within a specific gene set divided by the frequency in the rest of the *P*. *guiyangense* proteome; a chi-square test with p-value <0.05 was used for significance tests.

### Identification of putative elicitins and effectors

The elicitin domain (PF00964) was retrieved from the PFAM database [63], and then used to search against *P*. *guiyangense* proteome. Hits with E-value less than 10^−5^ were considered to be elicitin candidates. To distinguish elicitin (ELI) and elicitin-like (ELL) proteins, the previously known sequence features of the elicitin domain were used [28]. ELIs contain a highly conserved 98-amino acid domain with six cysteine residues and a typical cysteine spacing pattern. ELLs possess shorter or longer elicitin domains and sequences are more diverse.

For CRN effector prediction, well-characterized CRN proteins from *Ph*. *sojae* and *Ph*. *infestans* were obtained from a previous publication [14], and then were used to construct HMM profiles based on the LFLAK and HVLVVVP motifs. The HMM profiles were used to search against all possible proteins derived from six open reading frames of the genome. The resulting CRN candidates were manually examined for the presence of LFLAK and HVLVVVP motifs. After that, the validated CRN proteins were used to update the HMM profile, which was then used to search the protein database again for new candidates. To determine whether CRN candidates were full length or pseudogenes, we aligned the CRN candidates with previously characterized CRN proteins. If the CRN candidates shared similar sequence with known CRNs at the DNA level, but had an obvious frameshift mutation or earlier stop codon, they were considered to be pseudogenes. To predict secretion signals for CRN proteins, four signal peptide predictors including SignalP 3.0 (http://www.cbs.dtu.dk/services/SignalP-3.0/), SignalP 4.1 (http://www.cbs.dtu.dk/services/SignalP/), iPSORT (http://ipsort.hgc.jp/), PrediSi (http://www.predisi.de/), and one non-classical secretion signal predictor named SecretomeP 2.0 (http://www.cbs.dtu.dk/services/SecretomeP/), were utilized.

### Insect cell bioassays

*Spodoptera frugiperda* sf9 cell lines were cultured with sf-900™ III SFM medium (Gibco) at 27°C and 140 rpm in suspension flasks until they reached 2×10^6^ cells/mL. Sf9 cells were subcultured using fresh medium every 3 days. The mosquito C6/36 cell lines were cultured in Schneider’s Drosophila Medium (Gibco) with 10% Fetal Bovine Serum (Gibco) at 27°C and the culture medium was renewed every 3 days. Cell density was determined using a Countess Automated Cell Counter (Invitrogen) and cell viability was evaluated by staining with trypan blue exclusion dye.

To evaluate CRN protein toxicity, Sf9 cells were transfected with DNAs encoding CRN proteins. The ORFs of CRNs excluding the signal peptide were amplified, and CRNs without the signal peptide predicted by SignalP were amplified by excluding the N-terminal twenty-five amino acids. The PCR products were cloned into pIB/V5-His vector (Invitrogen) using ClonExpress II One Step Cloning Kit (Vazyme). Sf9 cell suspensions were seeded into 96-well plates (100 μL/well). After 24 h of incubation, cells were transfected with 0.2 ug plasmid DNA using the FuGENE HD Transfection Reagent (Promega) as described by the manufacturer, and six parallel wells were used in each group. Cell toxicity was detected by cell counting with the Cell Counting Kit-8 (CCK-8, Dojindo Laboratories Kumamoto, Japan) according to the manufacturer’s instructions. Briefly, after transfection of the Sf9 cells for 60 hours, 10 μL CCK8 solution was added to the cells. After the cells were incubated for 24 hours at 27°C, the absorbance was analyzed at 450 nm with a reference wavelength of 600 nm using SpectraMax M5 microplate reader. The cells receiving empty vector DNA were considered as 100% viable. Then, the cell viability rate was calculated as follows: Cell viability (%) = [(*As*-*Ab*)/(*Ac*-*Ab*)]×100%, where *As* represents the test well reading, *Ac* represents the empty vector well reading, and *Ab* represents a blank well reading. The data are expressed as the means ± SE based on at least three independent experiments. CRN constructs were compared to empty vector DNA using Student’s t-test. A difference with P < 0.05 was considered to be statistically significant.

To validate the toxicity of specific CRN proteins, the relevant CRN genes excluding the signal peptide were inserted in frame into the pET32a (+) vector (Novagen) by directional cloning between the BamHI and HindIII sites. The pET32a empty vector was used as a negative control. Cell growth and induction of expression were carried out as described in the pET system manual. Briefly, *Escherichia coli* BL21(DE3) strains were grown at 37°C to an OD600 of 0.6. At that time, 1 mM IPTG was added in order to induce protein expression at 18°C for 18 h. Induced cells were harvested by centrifugation at 4°C and washed three times with PBS buffer. The cells were resuspended in PBS buffer and lysed using short (3 s) ultrasonic bursts separated by 6 s intervals for 6 min. Crude protein extracts were centrifuged for 10 min at 12,000 rpm. Then the extracts were filtered with a 0.22 um filter. Adherent sf9 or C6/36 cell monolayers in 96-well plates were incubated with the protein extracts for 4 hours. Cell viability was assayed using the methods in the above paragraph, and at least three independent repeats were performed.

### Western blot analysis

Cells were lysed in ice-cold lysis buffer (Solarbio) for 10 min. Following this, samples were centrifuged at 12,000 g at 4°C for 5 min. The supernatants were collected and boiled with loading buffer at 100°C for 10 min. The samples were separated by 10% SDS-PAGE and transferred onto a polyvinylidene difluoride membrane (Millipore, Billerica, MA, USA). Membranes were blocked with 5% non-fat milk then incubated with anti-His primary antibody for 2 h. The membranes were washed with 0.1% Tween 20 in PBS and probed with IRDye 800CW-conjugated goat (polyclonal) anti-mouse IgG secondary antibodies for 1 h at room temperature. PVDF membranes were visualized using a scanner (LI-COR Odyssey) with excitation at 700 and 800 nm.

## Supporting information

S1 Fig *P*. *guiyangense* is highly virulent to mosquitoes. (A) The cumulative survival curves of *Aedes albopictus* (Left) and *Culex pipiens pallens* (Right) larvae after inoculation with 4 agar plugs (10 mm × 10 mm in size) of *P*. *guiyangense* mycelia. (B) *P*. *guiyangense* infected egg, larva, pupa and adult stages of mosquitoes. (C) *Cx*. *pipiens pallens* larvae prefer to ingest *P*. *guiyangense* mycelia even in the adequate food environment. *Cx*. *pipiens pallens* larvae were put in the culture dish containing mycelia of *P*. *guiyangense*, and *P*. *aphanidermatum* (control), V8 medium and mosquito food.
(TIF)

S2 Fig Validation of RNA-Seq results by qRT-PCR assay. The relative expression levels of the selected 18 genes were verified by qRT-PCR. Error bars represented the SD for three independent experiments. Gene name was shown on the top of each pair of images.
(TIF)

S3 Fig Phylogenetic analyses and observation of nuclear number. (A) Phylogenetic tree of *Cox* II genes among 35 *Pythium* species. (B) Phylogenetic tree of β-tubulin genes among 35 *Pythium* species. (C) High percentage of zoospores contained two nuclei in *P*. *guiyangense*. Observation of nucleus numbers in zoospores by staining with 4',6-diamidino-2-phenylindole (DAPI). The numbers in the brackets represent the percentage of two nuclei observed in one zoospore (a total of 500 zoospores) for each species.
(TIF)

S4 Fig Distribution of the core, conserved, and species-specific genes among *Pythium* species. The cladogram was constructed based on the tree shown in Fig 3A. The letter "A" with blue background represents animal host, and the letter "P" with green background represents plant host. The colored bars represent the numbers of core (present in all the seven genomes), conserved (present in two to six genomes) or species-specific (present only in own genome) genes for each species, which were determined using OrthoMCL.
(TIF)

S5 Fig Neighbor-joining tree of subtilisin proteases encoded from all the seven available *Pythium* genomes. The red branches represent the subtilisin-like proteases from *P*. *guiyangense*.
(TIF)

S6 Fig Expansion of kazal protease inhibitors in *P*. *guiyangense*. (A) Neighbor-joining tree of kazal protease inhibitors encoded by the seven available *Pythium* genomes. (B) Relative abundance of *P*. *guiyangense* transcripts encoding kazal protease inhibitors at 24 hpi infection versus mycelia. (C) Validation of transcriptional levels of 4 kazal protease inhibitor genes at different infection time points by qRT-PCR. Error bars represented the SD for three independent experiments.
(TIF)

S7 Fig Virulence assays of CRN proteins in insect cells. (A) Expression of CRN proteins in Sf9 cells was confirmed with western blot. (B) Expression of CRN proteins in Sf9 cells was confirmed by detecting the fluorescence signals. (C) Prokaryotic expression of selected CRN proteins confirmed by western blot analysis. (D) qRT-PCR analysis of CRN31 transcript levels at early infection time points. (E) qRT-PCR analysis of CRN28 transcript levels at early infection time points. Transcript levels are given relative to the internal standard *actin* gene. MY, mycelia.
(TIF)

S1 Video Accumulated mycelia were visible on the larva breathing tube while the larvae could still move at 3–4 dpi. (MP4)

S2 Video The video showed that *Cx*. *pipiens pallens* larvae readily ingested *P*. *guiyangense* mycelia.
(MP4)

S1 Table Comparison of the completeness of the *Pythium* genomes based on 248 CEGs. (DOC)

S2 Table Transcriptome sequencing data for *P*. *guiyangense*. (DOC)

S3 Table Transcriptional changes of potential pathogenesis-related genes. (DOC)

S4 Table Identification of synteny blocks and homologous gene pairs in *P*. *guiyangense*. (XLSX)

S5 Table Protein domains enriched in *P*. *guiyangense* species-specific genes. (DOC)

S6 Table Transcript level changes of kinase genes in *P*. *guiyangense*. (DOC)

S7 Table Comparison of selected plant cell wall degrading enzymes. (DOC)

S8 Table Information of all the CRN candidates in *P*. *guiyangense*. (XLSX)

## References

[1] Diniz DFA, de Albuquerque CMR, Oliva LO, de Melo-Santos MAV, Ayres CFJ. Diapause and quiescence: dormancy mechanisms that contribute to the geographical expansion of mosquitoes and their evolutionary success. Parasite Vector. 2017;10. 10.1186/s13071-017-2235-0 PMC5485599 28651558

[2] Scholte EJ, Knols BG, Samson RA, Takken W. Entomopathogenic fungi for mosquito control: a review. Journal of insect science. 2004;4:19 15861235 10.1093/jis/4.1.19 PMC528879

[3] Quiroz Velasquez PF, Abiff SK, Fins KC, Conway QB, Salazar NC, Delgado AP, et al Transcriptome analysis of the entomopathogenic oomycete *Lagenidium giganteum* reveals putative virulence factors. Applied and environmental microbiology. 2014;80(20):6427–36. 10.1128/AEM.02060-14 25107973 PMC4178636

[4] Butt TM, Greenfield BP, Greig C, Maffeis TG, Taylor JW, Piasecka J, et al *Metarhizium anisopliae* pathogenesis of mosquito larvae: a verdict of accidental death. PloS one. 2013;8(12):e81686 10.1371/journal.pone.0081686 24349111 PMC3862491

[5] Su X. A new species of *Pythium* isolated from mosquito larvae and its ITS region of rDNA. Mycosystema. 2006;25(4):523–8.

[6] Yu S, Duan S, Su X. A study on induction conditions for a trypsin-like protease (Pr2) of *Pythium guiyangense* Su. Journal of guiyang medical college. 2008;33(3):221–4.

[7] Zhao J, Su X. The genetic transformation of *Pythium guiyangense* mediated by *Agrobacterium tumefaciens*. Mycosystema. 2008;27(4):594–600.

[8] Kamoun S. Molecular genetics of pathogenic Oomycetes. Eukaryot Cell. 2003;2(2):191–9. 10.1128/EC.2.2.191-199.2003 12684368 PMC154851

[9] Saunders GA, Washburn JO, Egerter DE, Anderson JR. Pathogenicity of fungi isolated from field-collected larvae of the Western treehole mosquito, *Aedes sierrensis* (Diptera: Culicidae). Journal of invertebrate pathology. 1988;52(2):360–3. 3183417 10.1016/0022-2011(88)90148-6

[10] Larkin RP, English JT, Mihail JD. Effects of infection by *Pythium* spp on root-system morphology of alfalfa seedlings. Phytopathology. 1995;85(4):430–5.

[11] Krajaejun T, Khositnithikul R, Lerksuthirat T, Lowhnoo T, Rujirawat T, Petchthong T, et al Expressed sequence tags reveal genetic diversity and putative virulence factors of the pathogenic oomycete *Pythium insidiosum*. Fungal biology. 2011;115(7):683–96. 10.1016/j.funbio.2011.05.001 21724174

[12] Lamour KH, Mudge J, Gobena D, Hurtado-Gonzales OP, Schmutz J, Kuo A, et al Genome sequencing and mapping reveal loss of heterozygosity as a mechanism for rapid adaptation in the vegetable pathogen *Phytophthora capsici*. Molecular plant-microbe interactions: MPMI. 2012;25(10):1350–60. 10.1094/MPMI-02-12-0028-R 22712506 PMC3551261

[13] Levesque CA, Brouwer H, Cano L, Hamilton JP, Holt C, Huitema E, et al Genome sequence of the necrotrophic plant pathogen *Pythium ultimum* reveals original pathogenicity mechanisms and effector repertoire. Genome Biol. 2010;11(7):R73 10.1186/gb-2010-11-7-r73 20626842 PMC2926784

[14] Haas BJ, Kamoun S, Zody MC, Jiang RH, Handsaker RE, Cano LM, et al Genome sequence and analysis of the Irish potato famine pathogen *Phytophthora infestans*. Nature. 2009;461(7262):393–8. 10.1038/nature08358 19741609

[15] Rujirawat T, Patumcharoenpol P, Lohnoo T, Yingyong W, Kumsang Y, Payattikul P, et al Probing the phylogenomics and putative pathogenicity genes of *Pythium insidiosum* by oomycete genome analyses. Sci Rep. 2018;8(1):4135 10.1038/s41598-018-22540-1 29515152 PMC5841299

[16] Jiang RHY, de Bruijn I, Haas BJ, Belmonte R, Lobach L, Christie J, et al Distinctive expansion of potential virulence genes in the genome of the oomycete fish pathogen *Saprolegnia parasitica*. Plos Genet. 2013;9(6). 10.1371/journal.pgen.1003272 PMC3681718 23785293

[17] Bozkurt TO, Schornack S, Banfield MJ, Kamoun S. Oomycetes, effectors, and all that jazz. Curr Opin Plant Biol. 2012;15(4):483–92. 10.1016/j.pbi.2012.03.008 22483402

[18] Jiang RH, Tyler BM. Mechanisms and evolution of virulence in oomycetes. Annu Rev Phytopathol. 2012;50:295–318. 10.1146/annurev-phyto-081211-172912 22920560

[19] Huang S, Su x. Biological studies on *Pythium guiyangense*, a fungal pathogen of mosquito larvae. Mycosystema. 2007;26(3):380–8.

[20] Braun-Galleani S, Ortiz-Merino RA, Wu Q, Xu Y, Wolfe KH. *Zygosaccharomyces pseudobailii*, another yeast interspecies hybrid that regained fertility by damaging one of its MAT loci. Fems Yeast Res. 2018;18(7). 10.1093/femsyr/foy079 PMC6093378 30052970

[21] Bertier L, Leus L, D’hondt L, de Cock AWAM, Hofte M. Host adaptation and speciation through hybridization and polyploidy in *Phytophthora*. PloS one. 2013;8(12). 10.1371/journal.pone.0085385 PMC3873470 24386473

[22] Louis VL, Despons L, Friedrich A, Martin T, Durrens P, Casaregola S, et al *Pichia sorbitophila*, an interspecies yeast hybrid, reveals early steps of genome resolution after polyploidization. G3 (Bethesda). 2012;2(2):299–311. 22384408 10.1534/g3.111.000745 PMC3284337

[23] Choo JH, Hong CP, Lim JY, Seo JA, Kim YS, Lee DW, et al Whole-genome de novo sequencing, combined with RNA-Seq analysis, reveals unique genome and physiological features of the amylolytic yeast *Saccharomycopsis fibuligera* and its interspecies hybrid. Biotechnol Biofuels. 2016;9:246 10.1186/s13068-016-0653-4 27872659 PMC5106798

[24] Wang Y, Tang H, Debarry JD, Tan X, Li J, Wang X, et al MCScanX: a toolkit for detection and evolutionary analysis of gene synteny and collinearity. Nucleic Acids Res. 2012;40(7):e49 10.1093/nar/gkr1293 22217600 PMC3326336

[25] Adhikari BN, Hamilton JP, Zerillo MM, Tisserat N, Levesque CA, Buell CR. Comparative genomics reveals insight into virulence strategies of plant pathogenic oomycetes. PloS one. 2013;8(10). 10.1371/journal.pone.0075072 PMC3790786 24124466

[26] Hanks SK, Hunter T. Protein kinases 6. The eukaryotic protein kinase superfamily: kinase (catalytic) domain structure and classification. FASEB J. 1995;9(8):576–96. 7768349

[27] Rimphanitchayakit V, Tassanakajon A. Structure and function of invertebrate Kazal-type serine proteinase inhibitors. Dev Comp Immunol. 2010;34(4):377–86. 10.1016/j.dci.2009.12.004 19995574

[28] Jiang RHY, Tyler BM, Whisson SC, Hardham AR, Govers F. Ancient origin of elicitin gene clusters in *Phytophthora* genomes. Molecular biology and evolution. 2006;23(2):338–51. 10.1093/molbev/msj039 16237208

[29] Pemberton CL, Salmond GPC. The Nep1-like proteins—a growing family of microbial elicitors of plant necrosis. Mol Plant Pathol. 2004;5(4):353–9. 10.1111/j.1364-3703.2004.00235.x 20565603

[30] Torto TA, Li S, Styer A, Huitema E, Testa A, Gow NA, et al EST mining and functional expression assays identify extracellular effector proteins from the plant pathogen *Phytophthora*. Genome Res. 2003;13(7):1675–85. 10.1101/gr.910003 12840044 PMC403741

[31] Kwa MSG, de Maagd RA, Stiekema WJ, Vlak JM, Bosch D. Toxicity and binding properties of the *Bacillus thuringiensis* delta-endotoxin Cry1C to cultured insect cells. Journal of invertebrate pathology. 1998;71(2):121–7. 10.1006/jipa.1997.4723 9500946

[32] Bawin T, Seye F, Boukraa S, Zimmer JY, Raharimalala FN, Ndiaye M, et al Histopathological effects of *Aspergillus clavatus* (Ascomycota: Trichocomaceae) on larvae of the southern house mosquito, *Culex quinquefasciatus* (Diptera: Culicidae). Fungal biology. 2016;120(4):489–99. 10.1016/j.funbio.2016.01.002 27020151

[33] Lacey CM, Lacey LA, Roberts DR. Route of invasion and histopathology of *Metarhizium anisopliae* in *Culex quinquefasciatus*. Journal of invertebrate pathology. 1988;52(1):108–18. 3418133 10.1016/0022-2011(88)90109-7

[34] Zhang S, Xia YX, Kim B, Keyhani NO. Two hydrophobins are involved in fungal spore coat rodlet layer assembly and each play distinct roles in surface interactions, development and pathogenesis in the entomopathogenic fungus, *Beauveria bassiana*. Molecular microbiology. 2011;80(3):811–26. 10.1111/j.1365-2958.2011.07613.x 21375591

[35] Aw KMS, Hue SM. Mode of infection of *Metarhizium* spp. fungus and their potential as biological control agents. J Fungi (Basel). 2017;3(2). 10.3390/jof3020030 PMC5715920 29371548

[36] Greenfield BP, Lord AM, Dudley E, Butt TM. Conidia of the insect pathogenic fungus, *Metarhizium anisopliae*, fail to adhere to mosquito larval cuticle. R Soc Open Sci. 2014;1(2):140193 10.1098/rsos.140193 26064542 PMC4448906

[37] Bonants PJ, Hagenaar-de Weerdt M, Man In ’t Veld WA, Baayen RP. Molecular characterization of natural hybrids of *Phytophthora nicotianae* and *P*. *cactorum*. Phytopathology. 2000;90(8):867–74. 10.1094/PHYTO.2000.90.8.867 18944508

[38] Goss EM, Cardenas ME, Myers K, Forbes GA, Fry WE, Restrepo S, et al The plant pathogen *Phytophthora andina* emerged via hybridization of an unknown *Phytophthora* species and the Irish potato famine pathogen, *P*. *infestans*. PloS one. 2011;6(9):e24543 10.1371/journal.pone.0024543 21949727 PMC3174952

[39] Bidochka MJ, Melzer MJ. Genetic polymorphisms in three subtilisin-like protease isoforms (Pr1A, Pr1B, and Pr1C) from *Metarhizium* strains. Can J Microbiol. 2000;46(12):1138–44. 11142404 10.1139/w00-112

[40] Terra WR, Ferreira C. Insect digestive enzymes—properties, compartmentalization and function. Comp Biochem Phys B. 1994;109(1):1–62.

[41] Wu DD, Wang GD, Irwin DM, Zhang YP. A profound role for the expansion of trypsin-like serine protease family in the evolution of hematophagy in mosquito. Molecular biology and evolution. 2009;26(10):2333–41. 10.1093/molbev/msp139 19578155

[42] Morris MT, Coppin A, Tomavo S, Carruthers VB. Functional analysis of *Toxoplasma gondii* protease inhibitor 1. J Biol Chem. 2002;277(47):45259–66. 10.1074/jbc.M205517200 12228242

[43] Morris MT, Carruthers VB. Identification and partial characterization of a second Kazal inhibitor in *Toxoplasma gondii*. Mol Biochem Parasitol. 2003;128(1):119–22. 12706808 10.1016/s0166-6851(03)00051-3

[44] Milstone AM, Harrison LM, Bungiro RD, Kuzmic P, Cappello M. A broad spectrum Kunitz type serine protease inhibitor secreted by the hookworm *Ancylostoma ceylanicum*. J Biol Chem. 2000;275(38):29391–9. 10.1074/jbc.M002715200 10893410

[45] Telleria EL, de Araujo APO, Secundino NF, d’Avila-Levy CM, Traub-Cseko YM. Trypsin-like serine proteases in *Lutzomyia longipalpis*—expression, activity and possible modulation by *Leishmania infantum* chagasi. PloS one. 2010;5(5). 10.1371/journal.pone.0010697 PMC2872664 20502532

[46] Tian MY, Huitema E, da Cunha L, Torto-Alalibo T, Kamoun S. A Kazal-like extracellular serine protease inhibitor from *Phytophthora infestans* targets the tomato pathogenesis-related protease P69B. Journal of Biological Chemistry. 2004;279(25):26370–7. 10.1074/jbc.M400941200 15096512

[47] Ekchaweng K, Evangelisti E, Schornack S, Tian M, Churngchow N. The plant defense and pathogen counterdefense mediated by *Hevea brasiliensis* serine protease HbSPA and *Phytophthora palmivora* extracellular protease inhibitor PpEPI10. PloS one. 2017;12(5):e0175795 10.1371/journal.pone.0175795 28459807 PMC5411025

[48] Wawra S, Belmonte R, Lobach L, Saraiva M, Willems A, van West P. Secretion, delivery and function of oomycete effector proteins. Curr Opin Microbiol. 2012;15(6):685–91. 10.1016/j.mib.2012.10.008 23177095

[49] Kamoun S. A catalogue of the effector secretome of plant pathogenic oomycetes. Annu Rev Phytopathol. 2006;44:41–60. 10.1146/annurev.phyto.44.070505.143436 16448329

[50] Rosenblum EB, Poorten TJ, Joneson S, Settles M. Substrate-specific gene expression in *Batrachochytrium dendrobatidis*, the chytrid pathogen of amphibians. PloS one. 2012;7(11):e49924 10.1371/journal.pone.0049924 23185485 PMC3502224

[51] Kuo A, Kohler A, Martin FM, Grigoriev IV. Expanding genomics of mycorrhizal symbiosis. Front Microbiol. 2014;5:582 10.3389/fmicb.2014.00582 25408690 PMC4219462

[52] Olivera IE, Fins KC, Rodriguez SA, Abiff SK, Tartar JL, Tartar A. Glycoside hydrolases family 20 (GH20) represent putative virulence factors that are shared by animal pathogenic oomycetes, but are absent in phytopathogens. BMC Microbiol. 2016;16(1):232 10.1186/s12866-016-0856-7 27716041 PMC5053185

[53] Liu T, Ye W, Ru Y, Yang X, Gu B, Tao K, et al Two host cytoplasmic effectors are required for pathogenesis of *Phytophthora sojae* by suppression of host defenses. Plant physiology. 2011;155(1):490–501. 10.1104/pp.110.166470 21071601 PMC3075790

[54] Wang C, Shen D, Wang J, Chen Y, Dong Y, Tang Z, et al An AGC kinase, PgAGC1 regulates virulence in the entomopathogenic oomycete *Pythium guiyangense*. Fungal biology. 2019;123(1):87–93. 10.1016/j.funbio.2018.11.006 30654961

[55] Chin CS, Alexander DH, Marks P, Klammer AA, Drake J, Heiner C, et al Nonhybrid, finished microbial genome assemblies from long-read SMRT sequencing data. Nat Methods. 2013;10(6):563–+. 10.1038/nmeth.2474 23644548

[56] Li H, Durbin R. Fast and accurate short read alignment with Burrows-Wheeler transform. Bioinformatics. 2009;25(14):1754–60. 10.1093/bioinformatics/btp324 19451168 PMC2705234

[57] Walker BJ, Abeel T, Shea T, Priest M, Abouelliel A, Sakthikumar S, et al Pilon: an integrated tool for comprehensive microbial variant detection and genome assembly improvement. PloS one. 2014;9(11):e112963 10.1371/journal.pone.0112963 25409509 PMC4237348

[58] Parra G, Bradnam K, Korf I. CEGMA: a pipeline to accurately annotate core genes in eukaryotic genomes. Bioinformatics. 2007;23(9):1061–7. 10.1093/bioinformatics/btm071 17332020

[59] Stanke M, Steinkamp R, Waack S, Morgenstern B. AUGUSTUS: a web server for gene finding in eukaryotes. Nucleic Acids Res. 2004;32(Web Server issue):W309–12. 10.1093/nar/gkh379 15215400 PMC441517

[60] Korf I. Gene finding in novel genomes. BMC Bioinformatics. 2004;5:59 10.1186/1471-2105-5-59 15144565 PMC421630

[61] Krzywinski M, Schein J, Birol I, Connors J, Gascoyne R, Horsman D, et al Circos: an information aesthetic for comparative genomics. Genome Res. 2009;19(9):1639–45. 10.1101/gr.092759.109 19541911 PMC2752132

[62] Wang D, Zhang Y, Zhang Z, Zhu J, Yu J. KaKs_Calculator 2.0: a toolkit incorporating gamma-series methods and sliding window strategies. Genomics Proteomics Bioinformatics. 2010;8(1):77–80. 10.1016/S1672-0229(10)60008-3 20451164 PMC5054116

[63] Finn RD, Coggill P, Eberhardt RY, Eddy SR, Mistry J, Mitchell AL, et al The Pfam protein families database: towards a more sustainable future. Nucleic Acids Res. 2016;44(D1):D279–85. 10.1093/nar/gkv1344 26673716 PMC4702930

[64] Yin Y, Mao X, Yang J, Chen X, Mao F, Xu Y. dbCAN: a web resource for automated carbohydrate-active enzyme annotation. Nucleic Acids Res. 2012;40(Web Server issue):W445–51. 10.1093/nar/gks479 22645317 PMC3394287

[65] Rawlings ND, Barrett AJ, Bateman A. MEROPS: the database of proteolytic enzymes, their substrates and inhibitors. Nucleic Acids Res. 2012;40(Database issue):D343–50. 10.1093/nar/gkr987 22086950 PMC3245014

[66] Trapnell C, Pachter L, Salzberg SL. TopHat: discovering splice junctions with RNA-Seq. Bioinformatics. 2009;25(9):1105–11. 10.1093/bioinformatics/btp120 19289445 PMC2672628

[67] Trapnell C, Williams BA, Pertea G, Mortazavi A, Kwan G, van Baren MJ, et al Transcript assembly and quantification by RNA-Seq reveals unannotated transcripts and isoform switching during cell differentiation. Nat Biotechnol. 2010;28(5):511–5. 10.1038/nbt.1621 20436464 PMC3146043

[68] Feng J, Meyer CA, Wang Q, Liu JS, Shirley Liu X, Zhang Y. GFOLD: a generalized fold change for ranking differentially expressed genes from RNA-seq data. Bioinformatics. 2012;28(21):2782–8. 10.1093/bioinformatics/bts515 22923299

[69] Grabherr MG, Haas BJ, Yassour M, Levin JZ, Thompson DA, Amit I, et al Full-length transcriptome assembly from RNA-Seq data without a reference genome. Nat Biotechnol. 2011;29(7):644–52. 10.1038/nbt.1883 21572440 PMC3571712

[70] Thompson JD, Higgins DG, Gibson TJ. CLUSTAL W: improving the sensitivity of progressive multiple sequence alignment through sequence weighting, position-specific gap penalties and weight matrix choice. Nucleic Acids Res. 1994;22(22):4673–80. 7984417 10.1093/nar/22.22.4673 PMC308517

[71] Tamura K, Peterson D, Peterson N, Stecher G, Nei M, Kumar S. MEGA5: molecular evolutionary genetics analysis using maximum likelihood, evolutionary distance, and maximum parsimony methods. Molecular biology and evolution. 2011;28(10):2731–9. 10.1093/molbev/msr121 21546353 PMC3203626

[72] Li L, Stoeckert CJ Jr., Roos DS. OrthoMCL: identification of ortholog groups for eukaryotic genomes. Genome Res. 2003;13(9):2178–89. 10.1101/gr.1224503 12952885 PMC403725

