# Supplementary figures and images for: Infection mechanisms and putative effector repertoire of the mosquito pathogenic oomycete Pythium guiyangense uncovered by genomic analysis
Source: PLoS Genet. 2019 Apr 24;15(4):e1008116. doi: 10.1371/journal.pgen.1008116 (PMC6502433; doi:10.1371/journal.pgen.1008116)

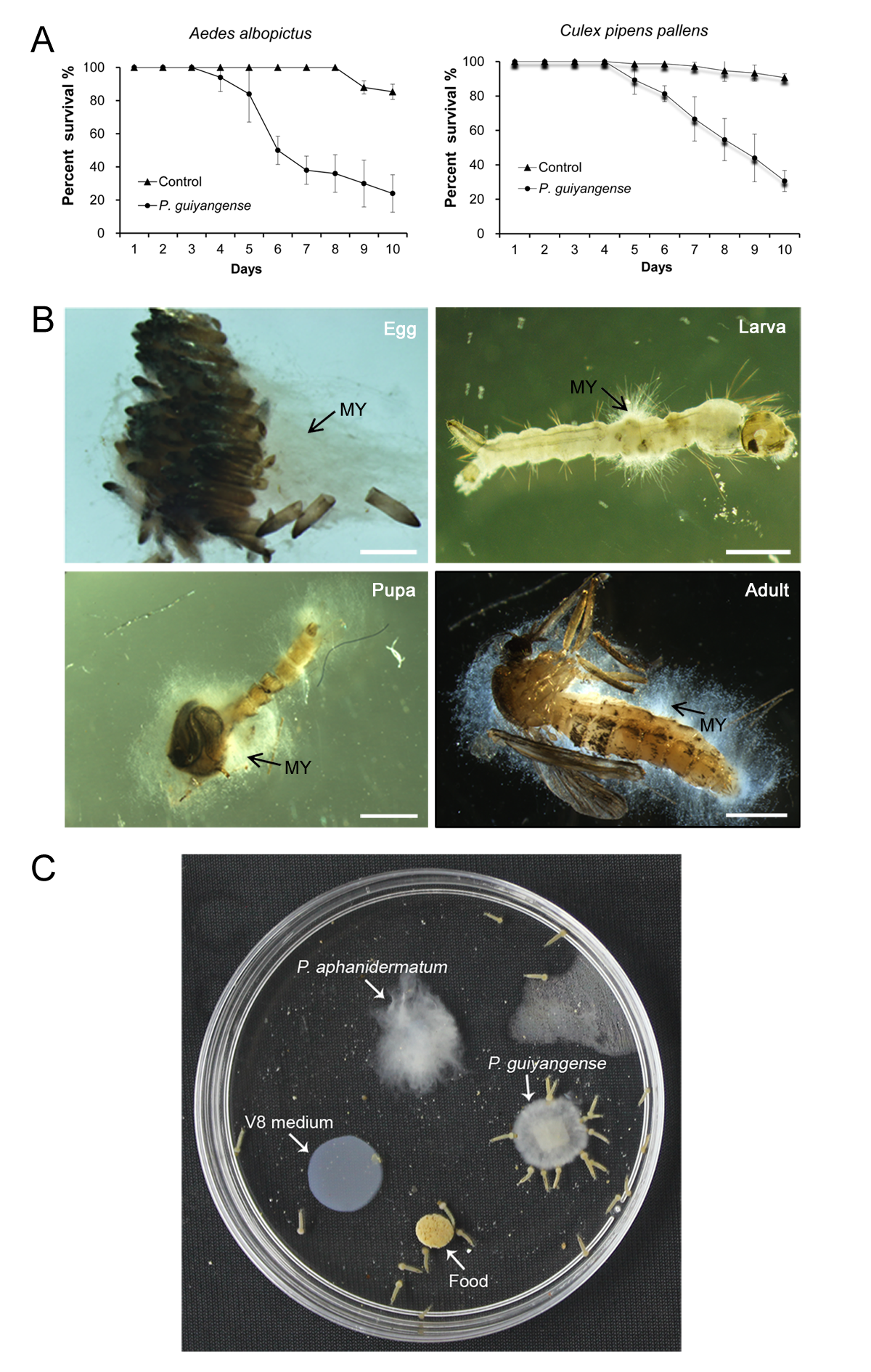

Supplement: S1 Fig — (A) The cumulative survival curves of Aedes albopictus (Left) and Culex pipiens pallens (Right) larvae after inoculation with 4 agar plugs (10 mm × 10 mm in size) of P. guiyangense mycelia. (B) P. guiyangense infected egg, larva, pupa and adult stages of mosquitoes. (C) Cx. pipiens pallens larvae prefer to ingest P. guiyangense mycelia even in the adequate food environment. Cx. pipiens pallens larvae were put in the culture dish containing mycelia of P. guiyangense, and P. aphanidermatum (control), V8 medium and mosquito food. (TIF) [file pgen.1008116.s001.tif]

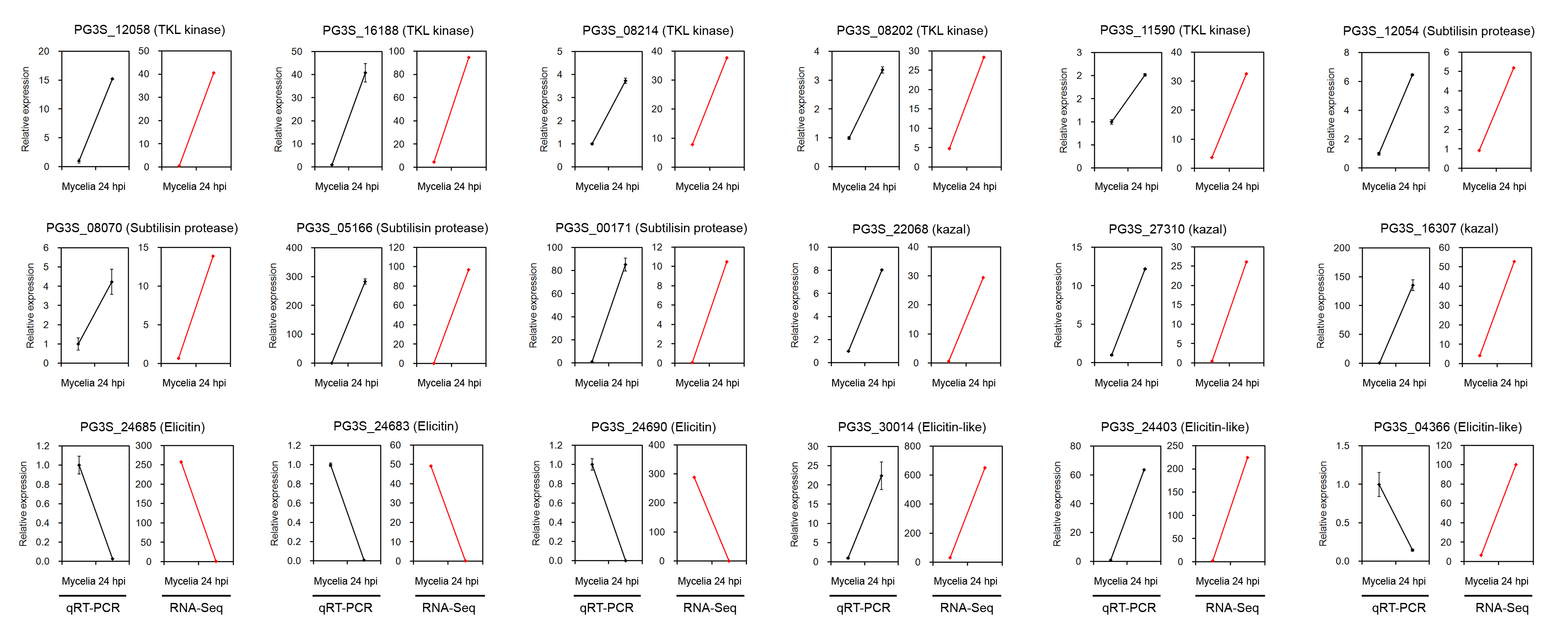

Supplement: S2 Fig — The relative expression levels of the selected 18 genes were verified by qRT-PCR. Error bars represented the SD for three independent experiments. Gene name was shown on the top of each pair of images. (TIF) [file pgen.1008116.s002.tif]

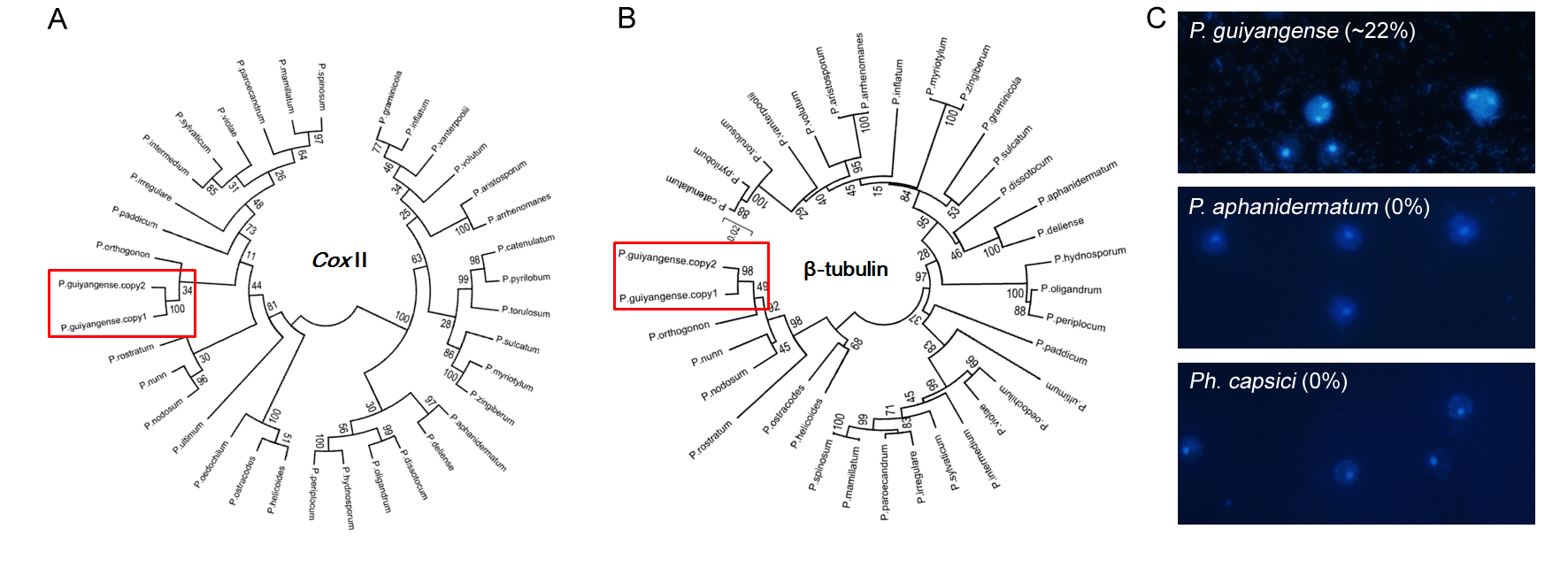

Supplement: S3 Fig — (A) Phylogenetic tree of Cox II genes among 35 Pythium species. (B) Phylogenetic tree of β-tubulin genes among 35 Pythium species. (C) High percentage of zoospores contained two nuclei in P. guiyangense. Observation of nucleus numbers in zoospores by staining with 4',6-diamidino-2-phenylindole (DAPI). The numbers in the brackets represent the percentage of two nuclei observed in one zoospore (a total of 500 zoospores) for each species. (TIF) [file pgen.1008116.s003.tif]

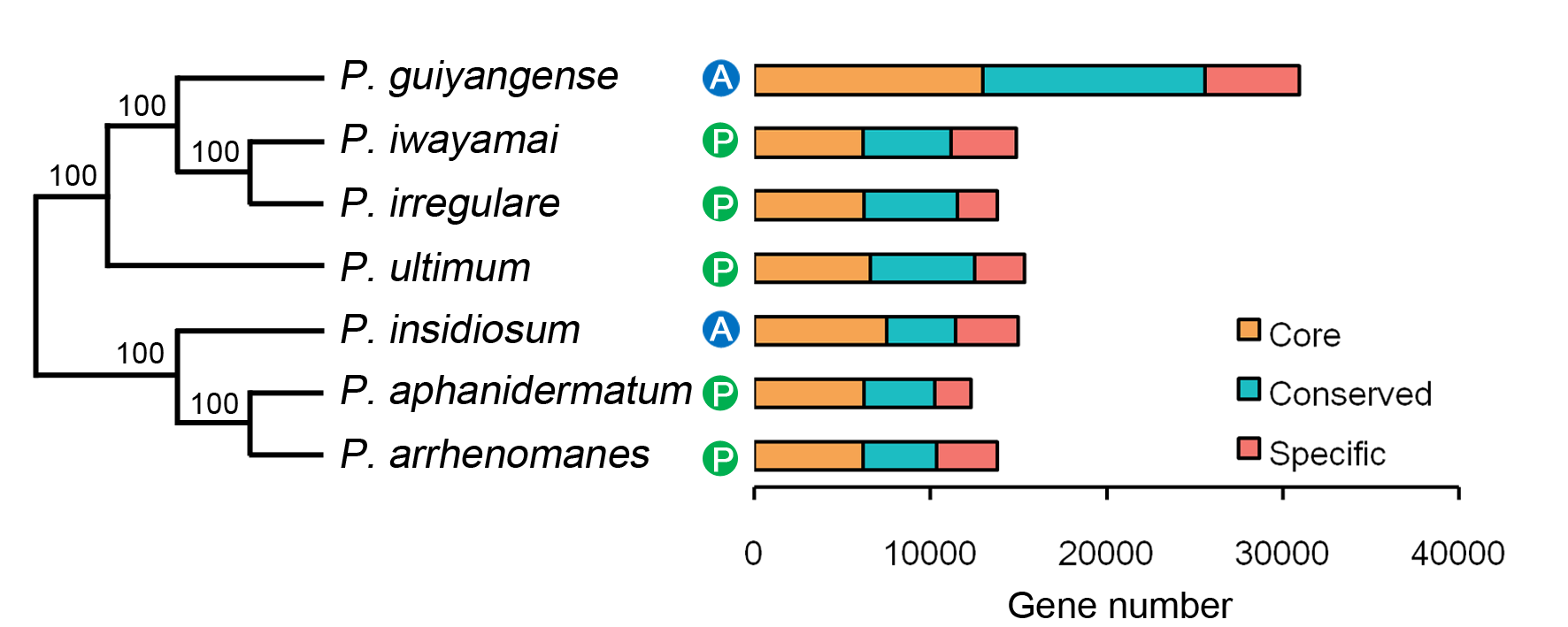

Supplement: S4 Fig — The cladogram was constructed based on the tree shown in Fig 3A. The letter "A" with blue background represents animal host, and the letter "P" with green background represents plant host. The colored bars represent the numbers of core (present in all the seven genomes), conserved (present in two to six genomes) or species-specific (present only in own genome) genes for each species, which were determined using OrthoMCL. (TIF) [file pgen.1008116.s004.tif]

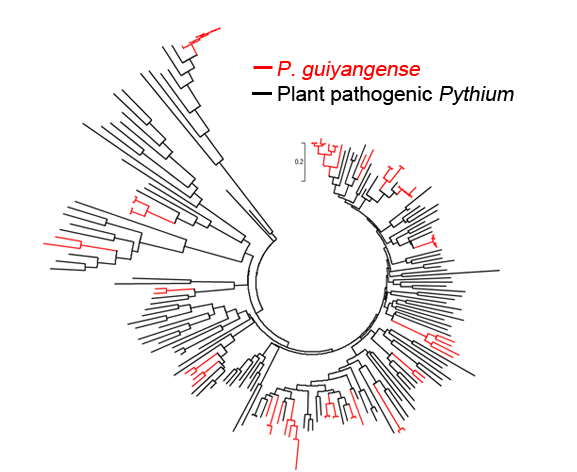

Supplement: S5 Fig — The red branches represent the subtilisin-like proteases from P. guiyangense. (TIF) [file pgen.1008116.s005.tif]

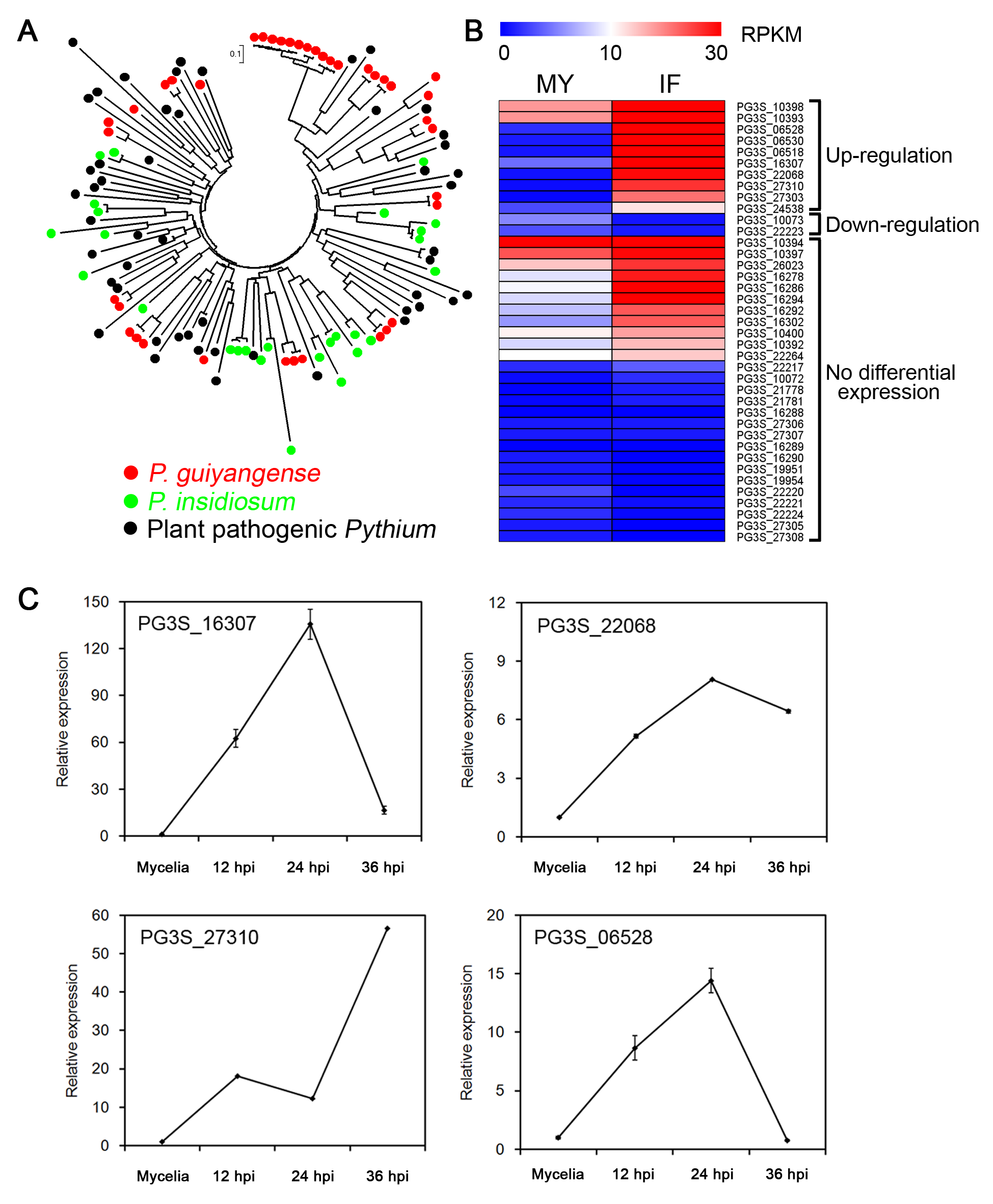

Supplement: S6 Fig — (A) Neighbor-joining tree of kazal protease inhibitors encoded by the seven available Pythium genomes. (B) Relative abundance of P. guiyangense transcripts encoding kazal protease inhibitors at 24 hpi infection versus mycelia. (C) Validation of transcriptional levels of 4 kazal protease inhibitor genes at different infection time points by qRT-PCR. Error bars represented the SD for three independent experiments. (TIF) [file pgen.1008116.s006.tif]

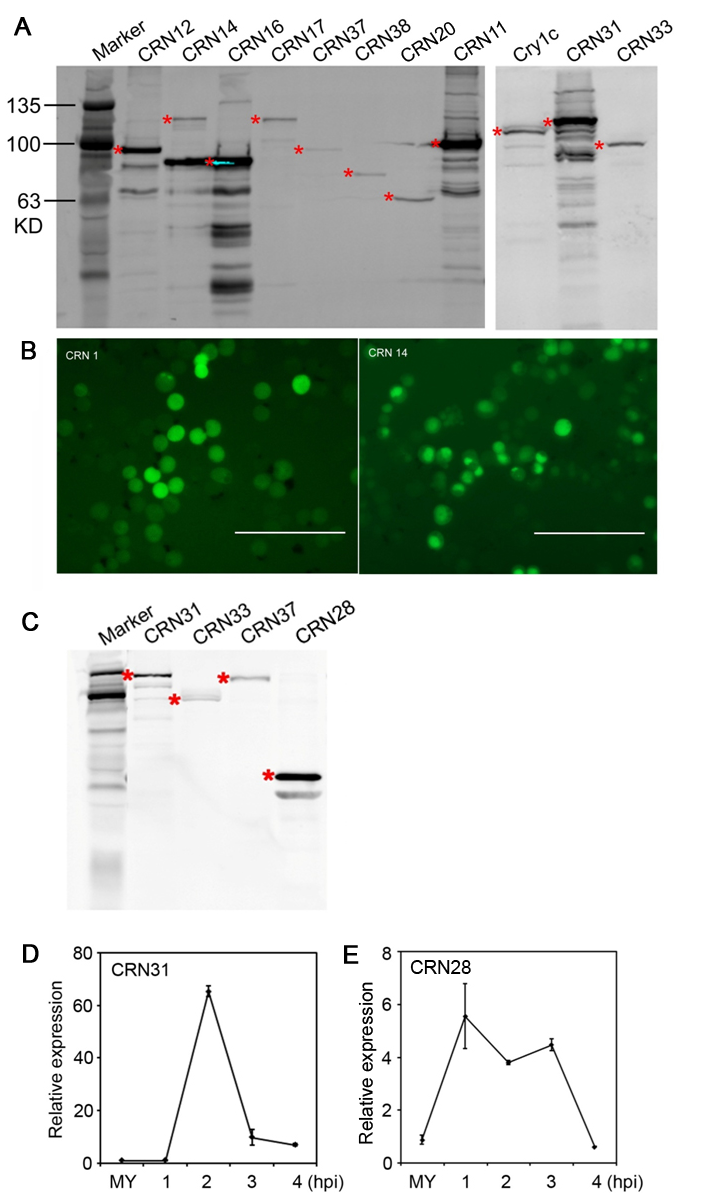

Supplement: S7 Fig — (A) Expression of CRN proteins in Sf9 cells was confirmed with western blot. (B) Expression of CRN proteins in Sf9 cells was confirmed by detecting the fluorescence signals. (C) Prokaryotic expression of selected CRN proteins confirmed by western blot analysis. (D) qRT-PCR analysis of CRN31 transcript levels at early infection time points. (E) qRT-PCR analysis of CRN28 transcript levels at early infection time points. Transcript levels are given relative to the internal standard actin gene. MY, mycelia. (TIF) [file pgen.1008116.s007.tif]
