# Supplementary material for: Infection mechanisms and putative effector repertoire of the mosquito pathogenic oomycete Pythium guiyangense uncovered by genomic analysis
Source: PLoS Genet. 2019 Apr 24;15(4):e1008116. doi: 10.1371/journal.pgen.1008116 (PMC6502433; doi:10.1371/journal.pgen.1008116)
Supplement: S1 Table — (DOC) [file pgen.1008116.s010.doc]

**S1 Table. Comparison of the completeness of the *Pythium* genomes based on 248 CEGs**

| Organism | Complete^a^ | | Partial^b^ | |
| --- | --- | --- | --- | --- |
|  | Protein number^c^ | Completeness^d^ | Protein number | Completeness |
| *P. guiyangense* | 242 | 97.58% | 244 | 98.39% |
| *P. ultimum* | 227 | 91.53% | 233 | 93.95% |
| *P. insidiosum* | 227 | 91.53% | 231 | 93.15% |
| *P. aphanidermatum* | 224 | 90.32% | 237 | 95.56% |
| *P. arrhenomanes* | 194 | 78.23% | 227 | 91.53% |
| *P. irregulare* | 234 | 94.35% | 237 | 95.56% |
| *P. iwayamai* | 215 | 86.69% | 227 | 91.53% |

^a^complete CEGs

^b^partial CEGs

^c^number of 248 CEGs present in the genome

^d^percentage of 248 CEGs present in the genome
