# Supplementary material for: Infection mechanisms and putative effector repertoire of the mosquito pathogenic oomycete Pythium guiyangense uncovered by genomic analysis
Source: PLoS Genet. 2019 Apr 24;15(4):e1008116. doi: 10.1371/journal.pgen.1008116 (PMC6502433; doi:10.1371/journal.pgen.1008116)
Supplement: S2 Table — (DOC) [file pgen.1008116.s011.doc]

**S2 Table. Transcriptome sequencing data for *P. guiyangense*.**

| RNA-Seq sample | Number of reads | Number of mapped reads | Number of expressed genes | Median RPKM | Highest RPKM |
| --- | --- | --- | --- | --- | --- |
| Mycelia | 90,173,360 | 75,717,869 | 21,418 | 8.78 | 6019 |
| Infection on mosquito larvae | 119,466,783 | 61,727,451 | 21,577 | 6.76 | 3605 |
