# Supplementary material for: Infection mechanisms and putative effector repertoire of the mosquito pathogenic oomycete Pythium guiyangense uncovered by genomic analysis
Source: PLoS Genet. 2019 Apr 24;15(4):e1008116. doi: 10.1371/journal.pgen.1008116 (PMC6502433; doi:10.1371/journal.pgen.1008116)
Supplement: S3 Table — (DOC) [file pgen.1008116.s012.doc]

**S3 Table. Transcriptional changes of potential pathogenesis-related genes**

|  | Total gene number | Up-regulated gene number | Down-regulated gene number |
| --- | --- | --- | --- |
| Kinases | 943 | 51 | 41 |
| TKL kinases | 440 | 37 | 15 |
| Kazal protease inhibitors | 39 | 10 | 2 |
| Proteases | 615 | 52 | 36 |
| Subtilisin proteases | 64 | 19 | 1 |
| Papain proteases | 68 | 2 | 7 |
| Elicitin genes | 20 | 0 | 9 |
| Elicitin-like genes | 91 | 28 | 14 |
| CRN genes | 38 | 1 | 4 |
