# Supplementary material for: Infection mechanisms and putative effector repertoire of the mosquito pathogenic oomycete Pythium guiyangense uncovered by genomic analysis
Source: PLoS Genet. 2019 Apr 24;15(4):e1008116. doi: 10.1371/journal.pgen.1008116 (PMC6502433; doi:10.1371/journal.pgen.1008116)
Supplement: S5 Table — (DOC) [file pgen.1008116.s014.doc]

**S5 Table. Protein domains enriched in *P. guiyangense* species-specific genes**

| **Domain accession** | **Domain description** | **Enrichment fold^a^** | **P-value^b^** |
| --- | --- | --- | --- |
| PF00162 | Phosphoglycerate kinase | 16.44 | 1.23E-09 |
| PF04525 | LURP-one-related | 10.96 | 6.33E-08 |
| PF00050 | Kazal domain | 9.95 | 4.66E-17 |
| PF13472 | SGNH hydrolase-type esterase domain | 7.05 | 2.46E-05 |
| PF02463 | RecF/RecN/SMC, N-terminal | 5.12 | 3.29E-04 |
| PF05225 | DNA binding HTH domain, Psq-type | 4.70 | 5.33E-03 |
| PF00433 | Pkinase_C | 4.17 | 9.69E-03 |
| PF00249 | SANT/Myb domain | 3.64 | 7.06E-17 |
| PF03083 | SWEET sugar transporter | 3.05 | 4.09E-04 |
| PF00063 | Myosin head, motor domain | 2.71 | 9.37E-04 |
| PF00964 | Elicitin | 2.71 | 8.79E-08 |
| PF02902 | Ulp1 protease family, C-terminal catalytic domain | 2.68 | 2.81E-04 |
| PF00024 | PAN_1 | 2.63 | 1.86E-02 |
| PF13365 | Trypsin_2 | 2.20 | 1.57E-02 |
| PF00240 | ubiquitin | 2.17 | 3.18E-02 |
| PF13843 | PiggyBac transposable element-derived protein | 6.04 | 4.05E-32 |
| PF03101 | FAR1 DNA binding domain | 4.00 | 1.10E-07 |
| PF02671 | Paired amphipathic helix | 18.79 | 2.14E-06 |
| PF13865 | Chromatin target of PRMT1 protein, C-terminal | 18.79 | 2.14E-06 |
| PF00538 | Linker histone H1/H5, domain H15 | 28.18 | 9.22E-06 |
| PF08031 | Berberine/berberine-like | 28.18 | 9.22E-06 |
| PF03941 | Inner centromere protein, ARK-binding domain | 3.00 | 1.46E-05 |
| PF04696 | Pinin/SDK/MemA protein | 3.00 | 1.46E-05 |
| PF06047 | NF-kappa-B-activating protein | 3.00 | 1.46E-05 |
| PF09713 | A_thal_3526 | 3.00 | 1.46E-05 |
| PF10172 | DDA1 | 3.00 | 1.46E-05 |
| PF12767 | SAGA-Tad1 | 3.00 | 1.46E-05 |
| PF12773 | DZR | 3.00 | 1.46E-05 |
| PF14661 | HAUS6_N | 3.00 | 1.46E-05 |
| PF14774 | FAM177 | 3.00 | 1.46E-05 |
| PF04078 | Rcd1 | 12.52 | 2.00E-05 |
| PF07648 | Kazal_2 | 3.97 | 3.30E-05 |
| PF00090 | Thrombospondin type-1 (TSP1) repeat | 14.09 | 1.33E-04 |
| PF00855 | PWWP domain | 7.51 | 3.95E-04 |
| PF04752 | Glutathione-specific gamma-glutamylcyclotransferase | 7.51 | 3.95E-04 |
| PF13696 | Zinc knuckle CX2CX3GHX4C | 4.70 | 6.42E-04 |
| PF00881 | Nitroreductase | 9.39 | 7.95E-04 |
| PF08244 | Glycosyl hydrolase family 32, C-terminal | 9.39 | 7.95E-04 |
| PF03999 | MAP65_ASE1 | 18.79 | 8.05E-04 |
| PF09807 | Elongator complex protein 6 | 18.79 | 8.05E-04 |
| PF08284 | RVP_2 | 4.03 | 1.99E-03 |
| PF04500 | Zinc finger, FLYWCH-type | 2.00 | 2.18E-03 |
| PF05808 | Podoplanin | 2.00 | 2.18E-03 |
| PF06390 | Neuroendocrine secretory protein | 2.00 | 2.18E-03 |
| PF08914 | Rap1 Myb domain | 2.00 | 2.18E-03 |

^a^Enrichment fold correspond to frequency of a given domain in species-specific genes over frequency in the rest of the proteome.

^b^Chi-square test, p-value < 0.05.
