# Supplementary material for: Infection mechanisms and putative effector repertoire of the mosquito pathogenic oomycete Pythium guiyangense uncovered by genomic analysis
Source: PLoS Genet. 2019 Apr 24;15(4):e1008116. doi: 10.1371/journal.pgen.1008116 (PMC6502433; doi:10.1371/journal.pgen.1008116)
Supplement: S6 Table — (DOC) [file pgen.1008116.s015.doc]

**S6 Table. Transcript level changes of kinase genes in *P. guiyangense***

|  | Number of total genes | Number of differentially expressed genes | Number of up-regulated genes | Number of down-regulated genes |
| --- | --- | --- | --- | --- |
| PKL kinases | 62 | 8 | 1 | 7 |
| CK1 kinases | 6 | 1 | 0 | 1 |
| STE kinases | 28 | 2 | 0 | 2 |
| TK/TKL kinases | 440 | 52 | 37 | 15 |
| CAMK kinases | 101 | 17 | 12 | 5 |
| CMGC kinases | 86 | 1 | 0 | 1 |
| AGC kinases | 93 | 4 | 1 | 3 |
| Atypical kinases | 8 | 0 | 0 | 0 |
| Other kinases | 119 | 7 | 0 | 7 |
