# Supplementary material for: Infection mechanisms and putative effector repertoire of the mosquito pathogenic oomycete Pythium guiyangense uncovered by genomic analysis
Source: PLoS Genet. 2019 Apr 24;15(4):e1008116. doi: 10.1371/journal.pgen.1008116 (PMC6502433; doi:10.1371/journal.pgen.1008116)
Supplement: S7 Table — (DOC) [file pgen.1008116.s016.doc]

**S7 Table. Comparison of selected plant cell wall degrading enzymes**

| Substrate | Plant cell wall degrading enzymes | *P. guiyangense*^a^ | *P. insidiosum* | *P. aphanidermatum* | *P. arrhenomanes* | *P. irregulare* | *P. iwayamai* | *P. ultimum* | *S. parasitica* |
| --- | --- | --- | --- | --- | --- | --- | --- | --- | --- |
| Pectin | PL1: Pectin and pectate lyase | 5 (10) | 1 | 7 | 2 | 5 | 2 | 12 | 2 |
| Pectin | PL3: Pectin and pectate lyase | 6 (12) | 5 | 14 | 3 | 6 | 2 | 15 | 0 |
| Pectin | PL4: Pectin and pectate lyase | 1 (2) | 0 | 0 | 2 | 2 | 2 | 2 | 0 |
| Pectin | GH28: polygalacturonase | 2 (4) | 0 | 5 | 3 | 2 | 1 | 4 | 2 |
| Pectin | GH43: endo-1,5-α-L-arabinosidase | 1 (2) | 1 | 2 | 2 | 2 | 1 | 3 | 0 |
| Pectin | GH53: endo-β-1,4-galactanase | 0 (0) | 0 | 0 | 1 | 0 | 0 | 2 | 0 |
| Pectin | GH78: α-L-rhamnosidase | 0 (0) | 0 | 1 | 0 | 1 | 1 | 0 | 0 |
| Cutin | CE5: Cutinase | 0 (0) | 0 | 9 | 6 | 0 | 0 | 1 | 1 |
| Xylan | GH10: endoxylanase | 0 (0) | 0 | 0 | 1 | 0 | 0 | 0 | 0 |
| Xylan | GH11: endoxylanase | 0 (0) | 0 | 1 | 1 | 0 | 0 | 0 | 0 |
| Xyloglucan | GH12: xyloglucan-β-1,4-D-endoglucanase | 0 (0) | 0 | 0 | 2 | 0 | 0 | 0 | 0 |
|  | GH20: β-hexosaminidase | 0 (0) | 0 | 0 | 0 | 0 | 0 | 0 | 2 |
|  | GH37: trehalase | 2 (4) | 2 | 2 | 2 | 2 | 2 | 3 | 1 |

^a^The numbers without brackets are unique gene numbers per haploid genome, while numbers in the brackets are total gene numbers.
